# Characterization of the *aodA*, *dnmA, mnSOD* and *pimA* genes in *Aspergillus nidulans*

**DOI:** 10.1038/srep20523

**Published:** 2016-02-05

**Authors:** Éva Leiter, Hee-Soo Park, Nak-Jung Kwon, Kap-Hoon Han, Tamás Emri, Viktor Oláh, Ilona Mészáros, Beatrix Dienes, János Vincze, László Csernoch, Jae-Hyuk Yu, István Pócsi

**Affiliations:** 1 Department of Biotechnology and Microbiology, Faculty of Science and Technology, University of Debrecen, Debrecen, Hungary; 2 Departments of Bacteriology and Genetics, The University of Wisconsin-Madison, Wisconsin, USA; 3 Department of Pharmaceutical Engineering, Woosuk University, Wanju, Republic of Korea; 4 Department of Botany, Faculty of Science and Technology, University of Debrecen, Debrecen, Hungary; 5 Department of Physiology, Faculty of Medicine, University of Debrecen, Debrecen, Hungary

## Abstract

Mitochondria play key roles in cellular energy generation and lifespan of most eukaryotes. To understand the functions of four nuclear-encoded genes predicted to be related to the maintenance of mitochondrial morphology and function in *Aspergillus nidulans*, systematic characterization was carried out. The deletion and overexpression mutants of *aodA*, *dnmA*, *mnSOD* and *pimA* encoding alternative oxidase, dynamin related protein, manganese superoxide dismutase and Lon protease, respectively, were generated and examined for their growth, stress tolerances, respiration, autolysis, cell death, sterigmatocystin production, hyphal morphology and size, and mitochondrial superoxide production as well as development. Overall, genetic manipulation of these genes had less effect on cellular physiology and ageing in *A. nidulans* than that of their homologs in another fungus *Podospora anserina* with a well-characterized senescence. The observed interspecial phenotypic differences can be explained by the dissimilar intrinsic stabilities of the mitochondrial genomes in *A. nidulans* and *P. anserina*. Furthermore, the marginally altered phenotypes observed in *A. nidulans* mutants indicate the presence of effective compensatory mechanisms for the complex networks of mitochondrial defense and quality control. Importantly, these findings can be useful for developing novel platforms for heterologous protein production, or on new biocontrol and bioremediation technologies based on *Aspergillus* species.

Fungal ageing and longevity are highly dependent on mitochondrial integrity and functions in both the yeast *Saccharomyces cerevisiae* and the filamentous fungus *Podospora anserina*^1^,^2^,^3^,^4^. The age-dependent instability and rearrangements of mitochondrial DNA (mtDNA) result in cease of the expression of mtDNA-encoded genes, the subsequent impairment of basically important mitochondrial functions like respiration and, finally, in the definite lifespan and senescence phenomenon typical of *P. anserina* cultures^3^,^4^,^5^,^6^,^7^,^8^. Because any failure in the maintenance of mitochondrial functions may result in the early onset of programmed cell death the elements of mitochondrial quality control system including the elimination of reactive oxygen species (ROS), damaged biomolecules and even impaired mitochondria (mitophagy) are intensively studied but still not fully understood in fungal biology^4^,^7^,^8^.

In *P. anserina*, disturbances in the supply and maintenance of respiratory chain proteins may lead to deleterious consequences and, hence, a strong connection between respiration and longevity should exist^6^,^9^,^10^. Paradoxically at first glance, the cytochrome C oxidase (COX) deficient mutant strains of *P. anserina* showed increased lifespan^6^,^10^,^11^ although *P. anserina* is an obligate aerobe fungus and, consequently, the loss of COX should be lethal. However, the fungus has the capability to induce an alternative oxidase (AOX) pathway to compensate for the impairment of the COX protein^6^,^10^,^11^.

Under normal physiological conditions, ROS are eliminated by an arsenal of antioxidative enzymes in fungal cells including superoxide dismutases, which convert superoxide to H_2_O_2_. Although these enzymes affect the mitochondrial ROS levels, mitochondrial stability, subsequent ageing and lifespan beneficially^12^,^13^ the overexpression of manganese superoxide dismutase encoded by *PaSod3* unexpectedly led to a reduced lifespan in *P. anserina*, and the deletion of the same gene increased the paraquat sensitivity but did not affect the peroxide tolerance and the lifespan of the fungus^14^. These data warn us that the roles of ROS in lifespan and ageing may be controversial under certain conditions^4^,^14^,^15^.

Mitochondrial dynamics (changes in the frequency of fission and fusion phenotypes) as part of the cellular quality control is an indispensable part of the cellular adaptation to rapidly changing environmental conditions including stress^4^,^7^,^16^. The fission of mitochondria is controlled by a dynamin-related protein, which is found in all eukaryotes including yeasts and filamentous fungi^7^,^17^,^18^. In *P. anserina*, mutations in the dynamin-related protein 1 encoding gene *PaDnm1* resulted in filamentous-like mitochondria and prolonged the lifespan of the fungus, *i.e.* some mycelia grew up to 1,000 days^18^. On the other hand, overexpression of *PaDnm1* increased disintegration of mitochondria even in young cultures^18^.

There is one more strategy to preserve the quality of mitochondrial proteins and, therefore, to increase the longevity of the mitochondria in fungi through the induction of the damaged and excess protein removal mechanisms. In these organelles, the soluble serine protease LON plays a central role in controlling degradation of the impaired proteins^4^,^7^. In *P. anserina*, the yeast LON ortholog *PaLon* protein is expressed at higher level in senescent cultures than in juvenile cultures. Overexpression of *PaLon* led to elevated resistance against external hydrogen-peroxide compared to a control strain, and increased amount of *PaLon* prolonged the lifespan of the cultures^15^. Cytochrome c oxidase deficient long-lived mutants of *P. anserina* was also characterized by increased LON activities^10^ and Adam *et al.*^19^ reported a decreased lifespan for the *PaLon* gene deletion mutant grown under physiological conditions and also demonstrated the contribution of PaLon protease to ascospore germination and sexual reproduction.

In this study, we characterize a group of nuclear-encoded genes that are throught to be important for mitochondrial functions in the model organism *Aspergillus nidulans*, a model fungus to many Aspergilli with outstanding industrial or biomedical importance^20^. The group of selected genes included homologs of functionally characterized fungal AOX alternative oxidase, DNM1 dynamin-related protein 1, MnSOD superoxide dismutase and the LON serine protease genes^14^,^21^,^22^. Notably, a more recent study by Li *et al.*^23^ managed to link oxidative stress, mitochondrial disfunction and subsequent apoptotic cell death to the spontaneous degeneration of *A. nidulans* maintained on artificial culture media, which adds a strong argument supporting the applicability of this study.

## Results

A series of *A. nidulans* mutants were generated through the deletion and overexpression of nuclear-encoded genes that were shown to be directly involved in the maintenance of the integrity and function of mitochondria in other fungi, and a number of key physiological, morphological and developmental phenotypes of the mutants were analyzed.

### Growth and stress challenges on nutrient agar plates

**Figure 1:**
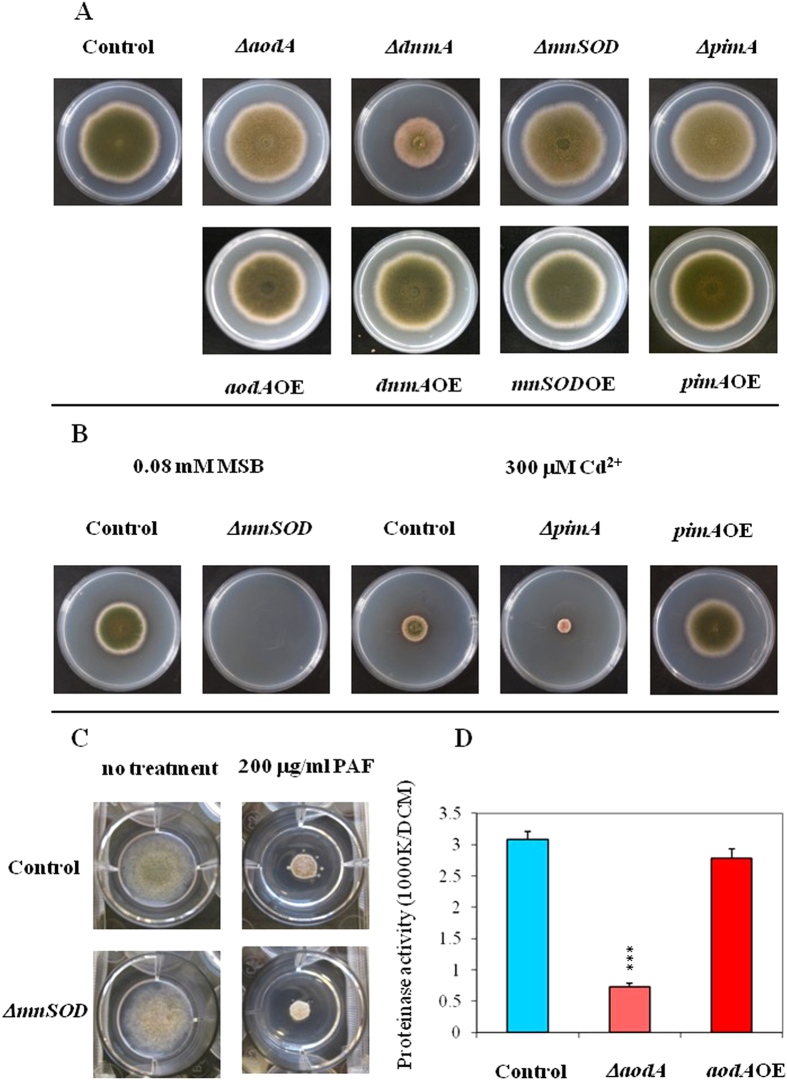
Comparison of the growth, stress sensitivities, cell death and autolysis of the control and mutant strains. Part A: Growth of control (THS30.3) and mutant strains on MNM agar plates. Part B: Selected stress sensitivity experiments with the control and the Δ*mnSOD*, Δ*pimA* and *pimA*OE strains. Part C: Growth inhibiting effect of the apoptosis-inducing antifungal protein PAF on the Δ*mnSOD* mutant in comparison to the control strain. Part D: Proteinase activities as autolysis markers^29^,^30^ recorded in carbon-starving (24 h) submerged cultures of the THS30.3 control and Δ*aodA* and *aodA*OE mutants. In proteinase assays, azocasein was used as substrate, and mean ± SD proteinase activity values calculated from three independent experiments are presented. The statistically significant difference calculated for the Δ*aodA* strain by the Student’s t test is marked with asterisks: ***p < 0.1%. Further phenotypes were not detected as shown in Supplementary Figs S1 and S3.

Various stress sensitivities of the mutant and control strains were tested on minimal nitrate medium (MNM) agar stress plates^24^. In general, fungal growth and stress sensitivities were hardly affected by the deletions and overexpressions of the mitochondrial function and morphology related genes and the phenotypic changes were rather sporadic (Fig. 1 and Supplementary Fig. S1). For example, the Δ*dnmA* mutant showed a reduced growth (approximately 40% decrease in colony diameter) even without any stress treatment in comparison to the control strain and the deletion of *mnSOD* displayed a remarkably high sensitivity to menadione exposure (Fig. 1B and Supplementary Fig. S1). The inactivation of *pimA* brought about decreased tolerance meanwhile overexpression of the same gene resulted in an increased tolerance to heavy metal stress initiated by CdCl_2_ (Fig. 1B and Supplementary Fig. S1). Inactivation of *aodA* did not influence the oxidative stress sensitivity of the fungus in accordance with previous findings reported by Suzuki *et al.*^25^ (2012). Quite surprisingly, only the Δ*mnSOD* mutant showed significantly increased sensitivity to PAF, an antifungal protein eliciting apoptotic cell death in sensitive fungi^26^. When employed at a concentration of 200 μg/ml PAF reduced the growth of the control and Δ*mnSOD* strains with approximately 60 and 75%, respectively (Fig. 1C and Supplementary Fig. S1).

### Growth and oxidative stress defense in submerged culture

Growth of the mutants was also characterized in submerged cultures by recording gains in the dry cell mass (DCM) (Supplementary Table S1). Complex media were inoculated with conidiospores and, after 16 h incubation, mycelia were washed to MNM medium supplemented with 2% glucose and also to glucose-free minimal medium. Although significant delays in biomass gains were observed in glucose supplemented cultures of the Δ*aodA*, *dnmA*OE (“OE” indicates “overexpression” mutant) and *mnSOD*OE strains and, to a lesser extent, with the Δ*dnmA* and Δ*pimA* gene deletion strains after 4 h incubation these differences disappeared when the incubation times were increased up to 10 h (Supplementary Table S1). It is noteworthy that the biomass values of the strains were comparable after 24 h carbon starvation in glucose-free culture medium independently of the genotypes with the exception of the Δ*aodA* strain where no significant autolytic loss of biomass was recorded. In addition, all strains reached similar biomass gains when they were transferred to MNM supplemented with 1% glucose, which indicates similar survival rates of the carbon-starving control and mutant strains (Supplementary Table S1).

To test the reactive species (RS) productions of the strains tested, mycelia grown in submerged cultures were stained by the fluorescent dye 2′,7′-dichlorofluorescin diacetate (Tables 1 and 2). DCF (2′,7′-dichlorofluorescein) production, which is proportional to RS generation^24^,^27^, was higher in glucose-supplemented cultures of the Δ*aodA* mutant incubated for 4 h but was lower when the fungus was incubated in glucose-free minimal medium for 24 h. Both the deletion and overexpression of *pimA* increased DCF levels in glucose-supplemented submerged cultures when compared to the control. It is noteworthy that similar tendency was also observed when the Δ*pimA* strain was incubated in glucose-free medium for 24 h. The overexpression of *mnSOD* also decreased the intracellular RS levels considerably in both glucose supplemented and glucose-free cultures. Although further minor phenotypes appeared in ∆*mnSOD* (in the presence of 2% glucose) and ∆*dnmA* (in the absence of glucose) cultures these changes were significant only at *p* < 5*%* levels (Tables 1 and 2).

To screen the oxidative stress defense systems of the mutants, a selection of antioxidant enzyme activities, including glutathione peroxidase (GPx), glutathione reductase (GR), catalase and superoxide dismutase (SOD) specific activities, were measured (Tables 1 and 2). In cultures supplemented with 2% glucose, the deletion of alternative oxidase increased the specific catalase, SOD and GR activities, while the GPx activity was lower than that found in the control strain. In the *aodA*OE mutant, GPx and GR levels increased significantly. The deletion of *dnmA* elevated the specific activities of all tested enzymes meanwhile the GPx activity decreased and, surprisingly, the catalase activity concomitantly increased significantly in the *dnmA*OE strain. Manipulation of MnSOD production affected the GR, catalase and, as expected, the SOD activities (Table 1). Deletion of *mnSOD* raised GR and catalase activities while overproduction of MnSOD lowered the activity of catalase but increased the SOD activity. Interestingly, high specific GPx, GR and catalase activities were found in the Δ*pimA* strain (Table 1).

In carbon-starving ageing cultures of the Δ*aodA* strain, both the specific catalase and SOD activities decreased meanwhile the *aodA*OE mutant displayed reduced GPx, GR, catalase and SOD activity levels. The overexpression of *dnmA* mitigated all enzyme activities tested but the deletion of *dnmA* did not influence the antioxidative enzyme activities at all. The overexpression of *mnSOD* resulted in lower GPx, catalase as well as SOD activities. In the *pimA*OE mutant, low specific GPx, GR, catalase and SOD activities were measured, and the specific catalase activities in the ∆*pimA* strain were of higher than those recorded in the control strain (Table 2).

### Production of sterigmatocystin and autolysis-related proteinase

As oxidative stress response has been connected to secondary metabolite production^28^, we determined the sterigmatocystin (ST) yields^24^ in carbon-starving submerged cultures of the mutants (Table 2, Supplementary Fig. S2). Importantly, the overexpression of *aodA* as well as the deletion of *aodA*, *dnmA* and *pimA* decreased ST productions meanwhile mycotoxin production increased considerably (more than twofold) in the *pimA*OE strain and, to a lesser extent, in the Δ*mnSOD* and *mnSOD*OE strains (Table 2; Supplementary Fig. S2).

We also determined the autolytic processes of the carbon-starving (16 + 24 h) submerged cultures by measuring proteinase activity accumulating in aging *A. nidulans* cultures^29^. Among our mutants, only the deletion of *aodA* alternative oxidase gene decreased significantly the specific autolytic proteinase activity which prevented any autolytic loss of dry cell mass (DCM) (Fig. 1D; Supplementary Fig. S3; Supplementary Table S1).

### Respiration

Mitochondria are the central organelles of respiration and, therefore, total, cytochrome c-dependent KCN-sensitive, alternative oxidase (AOX)-dependent {KCN-resistant, SHAM (salicylhydroxamic acid)-sensitive and remaining (KCN + SHAM resistant, *i.e.* resitant to the concomitant addition of KCN and SHAM) respirations were determined.

**Figure 2:**
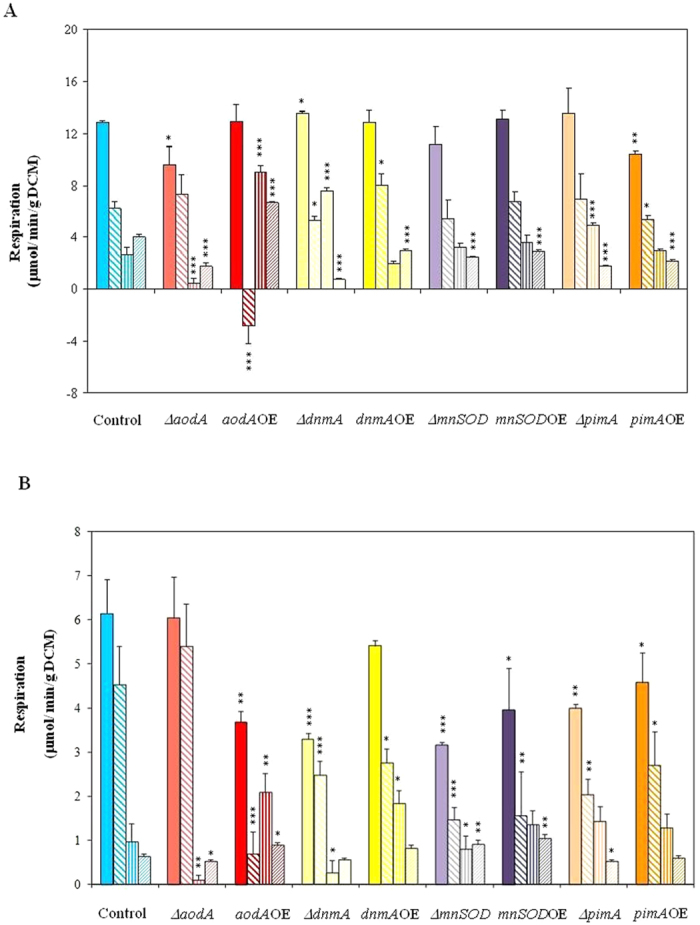
Respiration rates. Changes in total (■), KCN-sensitive (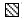) alternative oxidase (AOX; 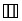) and KCN + SHAM-resistant respirations (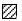) ( μmol/min/g DCM) recorded either in young cultures supplemented with 2% glucose (Part A) or in old, carbon starving cultures (Part B). KCN-sensitive and AOX-dependent respiration were calculated by substraction of KCN-resistant from total respiration and of KCN-SHAM from KCN respiration, respectively. In Part A, the addition of KCN even stimulated the respiration of the *aodA*OE strain and, hence, we got a virtual negative value for the KCN-sensitive respiration in this case. This paradoxical behavior can be explained with the considerably increased capacity of the AOX-dependent respiratory pathway similar to that observed before in the *P. anserina* AOX overexpression strain by Lorin *et al.*^6^. Mean ± SD values calculated from three independent experiments are presented. Statistically significant differences compared to the control determined by the Student’s t test are marked with asterisks: *p < 5%, **p < 1%, ***p < 0.1%. In all respiration rate assays, the THS30.3 strain was used as the control strain.

As shown in Fig. 2, all types of respirations were typically lower in carbon-starving cultures but these differences were highly unequal, *e.g.* 52.3, 27.1, 63.7 and 84% lower values were recorded for the total, cytochrome c-dependent, AOX-dependent and remaining respirations, respectively, when carbon-starving and glucose supplemented cultures of the THS30.3 control strain were compared (Fig. 2A,B). Very similar tendencies in total and KCN-resistant respirations were detected previously in carbon-starving *A. nidulans* cultures^30^.

In this study, in cultures supplemented with 2% glucose, manipulation of AOX production resulted in significant changes in the cyanide-resistant respiration. The deletion of *aodA* decreased while the overexpression of the same gene increased the cyanide-resistant as well as the KCN + SHAM-resistant (remaining) respirations of the fungus (Fig. 2A). It is noteworthy that the total respiration of the ∆*aodA* strain decreased in the presence of glucose meanwhile the respiration of the *aodA*OE strain even increased paradoxically resulting in a negative value KCN-sensitive respiration when the culture was supplemented with KCN (Fig. 2A). In carbon-starving cultures, similar tendencies were observed with some differences; meanwhile the total and the cytochrome c-dependent respirations were not affected in ∆*aodA* cultures they were considerably reduced in the *aodA*OE strain (Fig. 2B).

Furthermore, the deletion of *dnmA* significantly increased the total and the KCN-resistant AOX-dependent and decreased the remaining respirations of the cultures grown in the presence of 2% glucose (Fig. 2A). In ageing cultures, the gene deletion decreased considerably the total and the KCN-sensitive cytochrome c-dependent respirations (Fig. 2B). The KCN + SHAM resistant remaining respiration was significantly lower in both the Δ*dnmA* and the *dnmA*OE strains in the presence of glucose (Fig. 2A). In both the Δ*mnSOD* and *mnSOD*OE strains, the KCN + SHAM resistant respiration decreased in comparison to the control strain (Fig. 2A). In ageing cultures of the Δ*mnSOD* and *mnSOD*OE strains, the KCN + SHAM resistant respiration remained higher than that found in control cultures (Fig. 2B). Importantly, both the deletion and overexpression of *mnSOD* decreased the total and the KCN-sensitive cytochrome c-dependent respirations in ageing cultures (Fig. 2B) and a lower AOX-dependent respiration was also detected in the Δ*mnSOD* strain. The overexpression of *pimA* decreased the total, the KCN-sensitive cytochrome c-dependent and the KCN + SHAM resistant remaining respirations in the presence of glucose while the AOX-dependent respiration was increased and the remaining activity was decreased by Δ*pimA* mutation (Fig. 2A). In ageing cultures, the total and the cytochrome c-dependent respirations of the Δ*pimA* strain decreased meanwhile the respiration of the *pimA*OE strain was decreased only a lower level of significance (*p* < 5%) when compared to that of the THS30.3 control strain (Fig. 2B).

### Mitochondrial morphology

**Figure 3:**
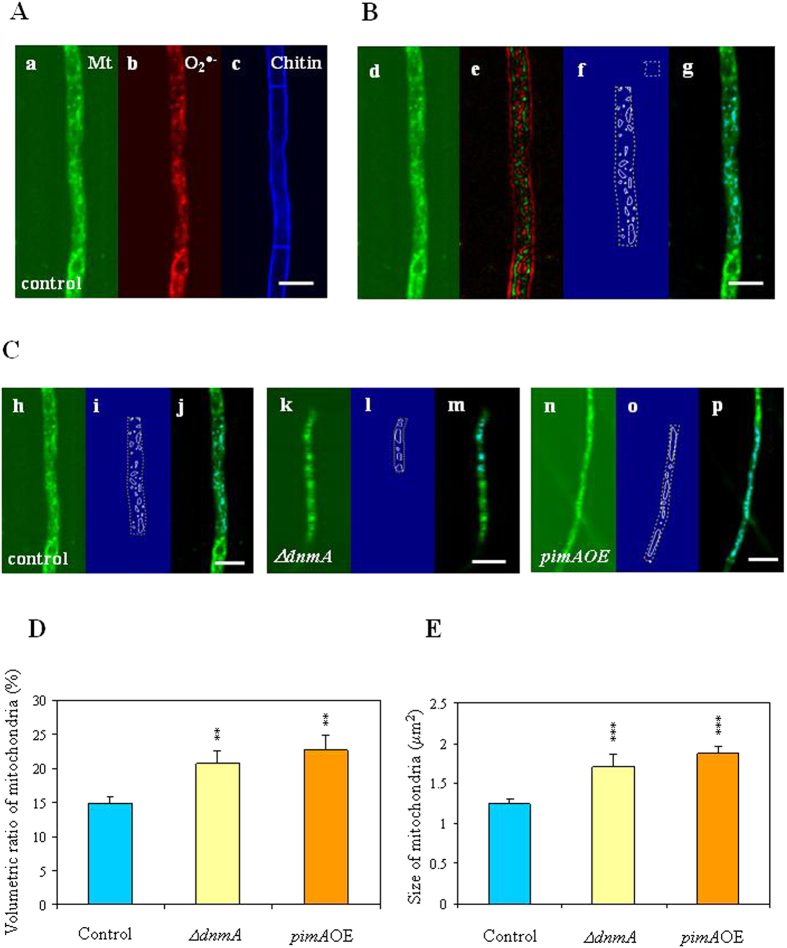
Visualization and characterization of mitochondria. Panel A: Three-channel confocal Z-stack images were taken where the green channel shows mitochondria **(Mt)** stained by MitoTracker Green (**a**), the red channel visualizes the superoxide (**O**_**2**_^**∙−**^) indicator dihydroethidium (**b**), while the blue channel (chitin staining by Calcofluor White) shows structure of the hypha and the boundaries for each segment (**c**)^23^,^60^. Panel B: Visualizing mitochondria-identifying these cell organelles. (**d**) Mitochondria stained by MitoTracker Green. (**e**) 2nd wavelet level of the 2D SWT of the same frame. (**f**) Contours of ROI (solid yellow line) and background (dashed yellow line) selected manually and mitochondria (solid white line) as provided by the automatic segmentation. (**g**) Identified mitochondria (cyan colored areas) superimposed to the denoised image. Panel C: Comparing mitochondrial morphology in the second hyphal segment of the THS30.3 control strain (**h–j**), Δ*dnmA* (**k–m**) and *pimA*OE strains (**n–p**). Markings on subpanels (**i**,**l** and **o**) correspond to those on subpanel (**f**). Markings on subpanels (**j**,**m** and **p**) correspond to those on subpanel (**g**). Scale bar: 10 μm. Panels D and E: Comparison of the volumetric ratio and size of mitochondria in the THS30.3 control, and the Δ*dnmA* and *pimA*OE mutant strains. Mean ± SD values calculated from four independent experiments are presented. Statistically significant differences determined by the Student’s t test are marked with asterisks: **p < 1%, ***p < 0.1%. No further phenotypes concerning mitochondrial morphology were observed (Supplementary Fig. S4).

We studied mitochondrial morphology, superoxide production and hyphal diameter in 20–24 h cultures of *A. nidulans* mycelia using MitoTracker Green, dihydroethidium and Calcofluor White stainings, respectively^23^,^31^ (Fig. 3A–C, Supplementary Fig. S4). Examining the volumetric ratio of the mitochondria inside the selected region of interest, we found significant differences between the control and the mutant strains only in two cases. Namely, the deletion of *dnmA* and the overexpression of *pimA* increased the volumetric ratio of mitochondria in the second hyphal segments from the apices (Fig. 3D; Supplementary Fig. S4). Importantly, meanwhile the numbers of these organelles did not change significantly in these mutants the sizes of mitochondria expanded considerably (Fig. 3E; Supplementary Fig. S4). It is worth noting that no significant differences were found in superoxide productions visualized by dihydroethidium staining irrespective of changes in the volumetric ratio and size of mitochondria (Supplementary Fig. S4; Supplementary Table S4) although these organelles are the main source of ROS and also the main targets of ROS-induced organelle damages^1^,^4^,^7^,^23^. Surprisingly, overexpression of *aodA*, *dnmA* and *pimA* but not that of *mnSOD* resulted in significantly thinner hyphae (with approximately 30–50% reductions in hyphal diameters) in comparison to the control strain (Supplementary Fig. S4E).

### Cleistothecia formation and asexual sporulation, viability of conidiospores

**Figure 4:**
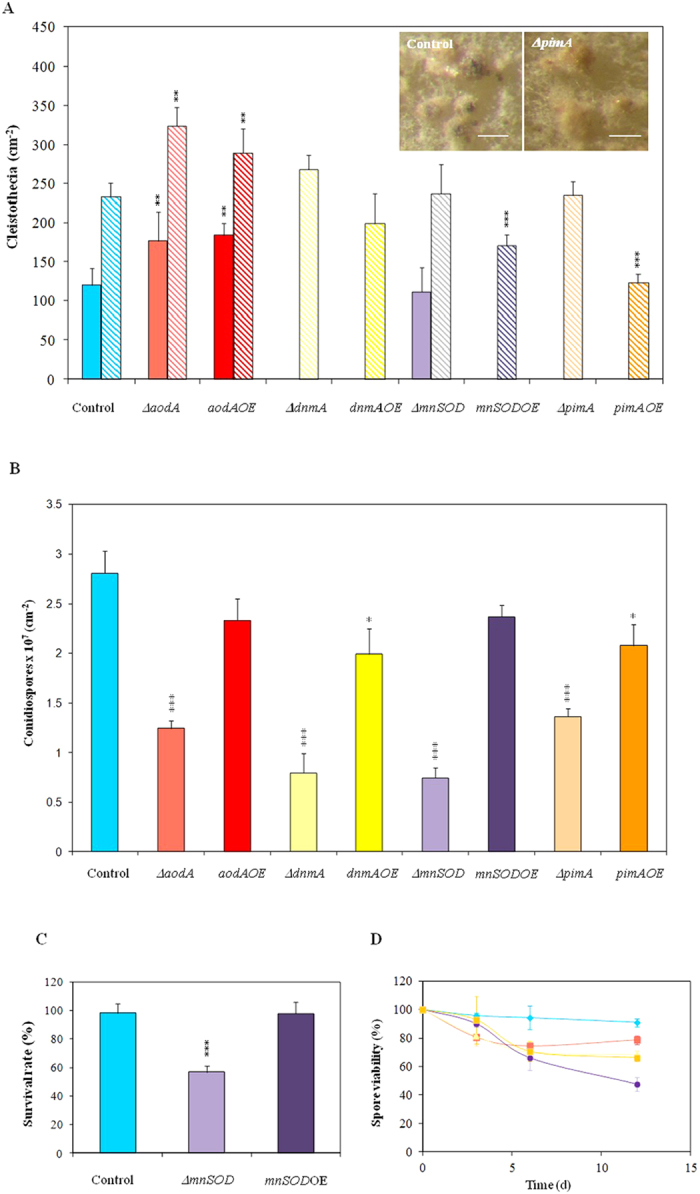
Cleistothecia and conidiospore productions and viability of conidia. Part A: Cleistothecia productions observed after 5 (■) and 8 d (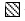) incubations. Both the deletion and overexpression of *aodA* stimulated meanwhile the overexpression of *mnSOD* and of *pimA* hindered the formation of fruiting bodies. Photographs show 8 d cleistothecia of the control (THS30.3) and Δ*pimA* strains. Note that cleistothecia of the Δ*pimA* strain were sterile. (Scale bars: 200 μm). Part B: Conidiospore productions. All gene deletion strains and the *pimA*OE overexpression strain produced less conidiospores than the control strain. Part C: Heat stress sensitivity of the Δ*mnSOD* conidia. Conidia without heat treatment (50 °C for 10 min) were used as control^32^. As shown in Supplementary Fig. S5, only conidia of the Δ*mnSOD* strain were sensitive to heat stress. Part D: Decreasing asexual spore viabilities during storage at 4 °C. Symbols represent the following strains: 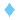, THS30.3 (control strain), 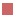, ∆*aodA*, 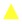, ∆*dnmA,* 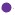, ∆*mnSOD* and, 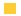, ∆*pimA*. The spore viabilities of all gene deletion strains were significantly lower (*p* < 5% at 6 and 12 d incubation times) than that of the THS30.3 control strain. Note that no significant decreases in the conidiospore viabilities were recorded in the gene overexpression strains and, hence, these data are not shown here for clarity. In Parts A-C, mean ± SD values calculated from three independent experiments are presented. Significant differences determined by the Student’s t test are marked with asterisks: **p < 1%, ***p < 0.1%. In Part D, per cent decreases in the spore viabilities (mean ± SD values calculated from three independent experiments) are presented.

We also quantified fruiting body formation and conidiospore production in all mutants^24^,^32^. Both the deletion and overexpression of *aodA* increased the number of cleistothecia meanwhile the relative numbers of fruiting bodies were less in the *pimA*OE and *mnSOD*OE strains than those found in control cultures (Fig. 4A). The maturation of cleistothecia has also been accelerated in the Δ*aodA* and *aodA*OE mutants, and completely matured cleistothecia were observed after 4 d incubation in the *aodA*OE strain (175 ± 32 cm^−2^ compared to the control 2.2 ± 0.1 cm^−2^; Supplementary Fig. S6). Surprisingly, the Δ*pimA* mutant was unable to produce ascospores even after 12 d incubation although the number of cleistothecia was within the normal range. Considering asexual sporulation, the deletion of each gene tested resulted in considerably reduced conidiospore formation. The relative numbers of conidiospores were also slightly reduced by overexpression of *dnmA* and *pimA* (Fig. 4B).

We also tested the viability of the conidiospores under different stress conditions^32^,^33^. Incubation of spores of the Δ*mnSOD* mutant at 50 °C for 10 min resulted in an appr. 50% reduction in viability meanwhile the asexual spores of the control and other genetically modified strains were not affected by thermal stress at all (Fig. 4C and Supplementary Fig. S5). Short-term storage as well as long-term storage (3, 6 and 12 d) of the conidiospores at 4 °C reduced drastically the viability of the Δ*mnSOD*, and also decreased significantly the viabilities of the Δ*aodA*, Δ*dnmA* and Δ*pimA* asexual spores (Fig. 4D).

## Discussion

Mitochondria are the powerhouses of the cells in the overwhelming majority of eukaryotes including fungi^34^. As a major source of endogenous ROS in aerobe organisms, mitochondria are continuously subjected to oxidative damages. Thus, not surprisingly, the ROS-elicited decay of mitochondrial functions is considered as a leading cause for cell ageing processes and, ultimately, the onset of programmed cell death^1^,^4^. Although mitochondrial ageing and senescence is an intensively studied area in fungal biology, our current knowledge in this important field is limited mostly to two species, the baker’s yeast *S. cerevisiae* and the filamentous fungus *P. anserina*^1^,^2^,^3^,^4^.

**Figure 5:**
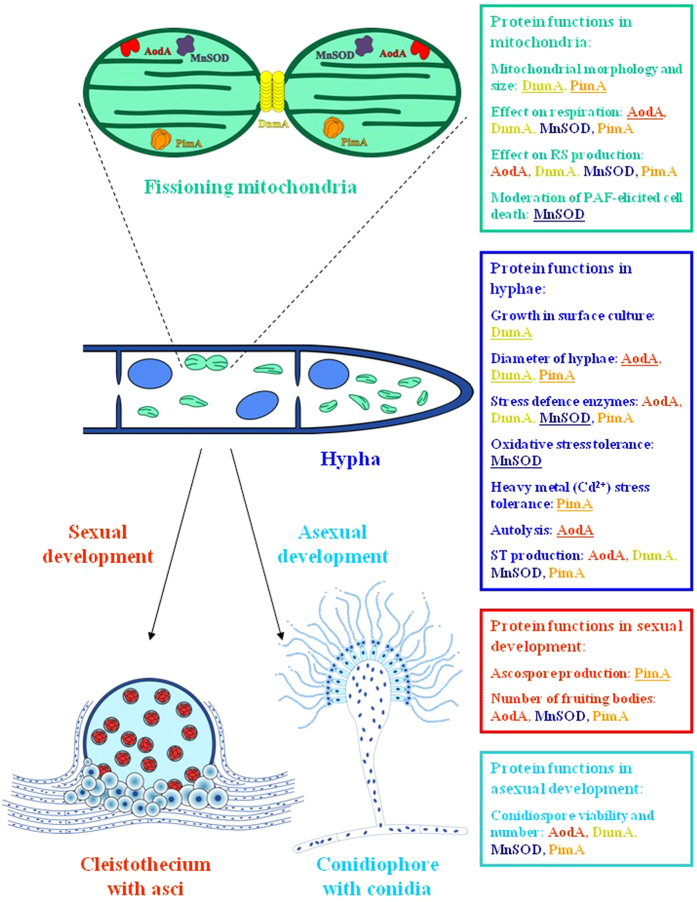
Summary of functions of AodA, DnmA, MnSOD and PimA in *A. nidulans*. Major functions revealed by the modulation of mitochondrial morphology and functions *via* the deletion and overexpression of the genes *aodA*, *dnmA*, *mnSOD* and *pimA* are summarized. Schematic mitochondrial, hyphal, cleistothecial and conidiophore structures are shown. In the dividing mitochondria, hypothetical protein structures and shapes are presented, which are based on homologous proteins characterized in various species (© Ibolya Pócsi; for further details see Supplementary Note S1).

In this study, we aimed at the functional investigation of four nuclear-encoded *A. nidulans* genes, whose homologs are associated with mitochondrial function and morphology, and have been found to influence ageing and longevity in *P. anserina*^14^,^21^,^22^. The *aodA*, *dnmA*, *mnSOD* and *pimA* genes predicted to encode alternative oxidase, dynamin-related protein, manganese superoxide dismutase and LON protease, respectively, were genetically manipulated, and a number of physiological, morphological and developmental features of the mutants were examined. Altogether 82 phenotypes were observable, which confirmed the importance of mitochondrial proteins in the maintenance of cellular and mitochondrial morphology and functions as well as in sexual and asexual developments (Fig. 5; Supplementary Note S1). Importantly, a decreased production and viability of conidiospores were observed commonly in all gene deletion mutants (Fig. 4; Supplementary Table S4), suggesting that these nucleus-encoded genes play a role in sporogenesis and the integrity of conidia. On the other hand, many phenotypes were sporadic and randomly distributed among the gene deletion and overexpression strains (Supplementary Table S4).

We then compared the phenotypes of relevant gene deletion and overexpression strains in *P. anserina*^6^,^14^,^15^,^18^,^19^,^21^ and *A. nidulans* as summarized in Table 3. Up to 26 features were chosen and the respective phenotypes were placed in one of the following four groups: (i) the same or similar phenotypes were described in the relevant *P. anserina* and the *A. nidulans* mutants, (ii) phenotype was observed in *P. anserina* but not in the corresponding *A. nidulans* mutant, (iii) phenotype was observed in *A. nidulans* but not in the corresponding *P. anserina* mutant and (iv) opposite phenotypes were found in the *P. anserina* and *A. nidulans* mutants (Table 3). The distribution of the features between these groups (6:12:7:1) showed that approximately the half (46.1%) of the phenotypes reported in the *P. anserina* strains were not observable in the corresponding *A. nidulans* mutants (Table 3). These findings clearly indicated that genetic modifications of these four genes have less impact on the cellular physiology and lifespan of *A. nidulans* compared to *P. anserina*^6^,^14^,^15^,^18^,^19^,^21^ (Table 3). Furthermore, eight phenotypes (30.8%) were either present only in *A. nidulans* or opposite to that recorded in *P. anserina* (Table 3), shedding light on further differences in their roles between the two ascomycetous filamentous fungi. These suggest a clear limit in using *P. anserina* as a model for predicting mitochondrial functions, life-span and longevity. This implies that the evolutionary divergence between *P. anserina* and other fungal species of interest^20^ makes the application of *Podospora*-based models on ageing and senescence^10^,^11^,^22^ quite difficult when important questions like strain degeneration processes and the control of lifespan are addressed in industrially and biomedically important filamentous fungi like the Aspergilli^23^.

Because only nuclear-encoded genes thought to have significant impacts on mitochondrial physiology and morphology were selected for genetic studies in *A. nidulans*, and their orthologs have been functionally characterized in *P. anserina*^6^,^14^,^15^,^18^,^19^,^21^, one may raise the important question that how the evolutionary distance between these two species influenced the unevenly emerging phenotypes of the mutants (Table 3). One option could be the divergent evolution of the mitochondrial gene contents through distinct species-specific gene transfer events to the nucleus^35^. However, the *P. anserina* mitochondrial genome harbors the same protein-coding potential except for the ATPase 9 gene as found before in *Neurospora crassa* and *A. nidulans*^36^. Therefore, we should consider another option, the significant differences in the size, organization, stability and intraspecies polymorphism of the *P. anserina* and *A. nidulans* mtDNAs^5^,^36^,^37^,^38^,^39^,^40^,^41^,^42^,^43^. We speculate that the primary reason for the phenotypic differences between *P. anserina*^6^,^14^,^15^,^18^,^19^, and *A. nidulans* would be due to the intrinsic instability of the *P. anserina* mtDNA.

Importantly, Li *et al.*^23^ found a causal connection between the spontaneous formation of fluffy sectors and mitochondrial dysfunctions in *A. nidulans* cultures maintained on potato dextrose agar. Although a linkage of culture degeneration to programmed cell death was demonstrated, the fluffy sector culture could be maintained further without any growth arrest or any sign of *P. anserina*-like senescence probably owing to compensatory mechanisms like the induction of stress defence elements including heat shock proteins, anti-apoptotic factors and DNA repair proteins^23^.

As mentioned above, these interesting phenotypes have also shed light on the remarkable importance of mitochondria in the developmental processes in filamentous fungi, especially concerning conidiogenesis and the preservation of the viability of conidia under storage at 4 °C (Fig. 4; Table 3). Further basic questions can include the elucidation of how the various upstream and downstream regulatory elements controlling development, stress, and metabolism in *A. nidulans*, such as ‘velvet’ proteins^44^,^45^, SskA and SrrA response regulators^32^,^33^, SakA mitogen-activated protein kinase^46^ and AtfA transcription factor^47^,^48^ contribute to the quality control of mitochondria^4^,^7^, and understanding how changes in the quality of mitochondrial functions will be sensed and how these signals are channeled towards regulatory elements by specific signaling pathways^49^,^50^.

It is worth noting that some observations presented in this paper also seem to be of great value from the biotechnologist’s point of view. For example, the deletion of *aodA* resulted in reduced production of proteinase and ST in *A. nidulans* (Table 2, Fig. 1D; Supplementary Figs S2 and S3), which may be suitable for heterologous protein production^51^. The *A. nidulans* Δ*aodA* mutant maintains satisfactory antioxidative defence even in the presence of endogenous oxidative stress (Supplementary Fig. S1), further suggesting the utility of novel Δ*aodA*-based *Aspergillus* industrial platforms for heterologous protein production. Interestingly, ST production was also decreased signifincantly *via* deletions of *dnmA* and *pimA* as well as through the overexpression of *aodA* (Table 2).

It is also remarkable that the overexpression of *pimA* resulted in the emergence of a new, heavy metal (Cd^2+^) tolerant *A. nidulans* strain, *pimA*OE (Fig. 1B, Supplementary Fig. S1), which could be beneficial when *Aspergillus*-based environmental technologies are elaborated^52^. Importantly, both asexual and sexual developments responded to the manipulation of the selected genes in many cases, and formation of cleistothecia was positively affected by either deletion or overexpression of *aodA* (Fig. 4, Supplementary Fig. S6). These observations on asexual and sexual sporulations may also be important when the potential industrial applications of genetically engineered *Aspergillus* strains are considered^53^.

Future studies are definitely needed to answer the intriguing questions whether these observations are limited to *A. nidulans* or are applicable and exploitable in other Aspergilli as well, *e.g.* in the biological control of toxigenic fungi^28^, in the bioremediation of heavy metal polluted areas^52^ in combating *Aspergillus* strain degenerations^23^.

To sum it up, the manipulation of four nuclear-encoded genes thought to be important for mitochondrial functions shed light on a number of physiological, morphological and developmental roles played by these genes in *A. nidulans* (Fig. 5; Supplementary Note S1). The remarkable differences observed between the phenotypes of the relevant *A. nidulans* and *P. anserina* mutants (Table 3; Supplementary Table 3) suggest that *A. nidulans*-based platforms should also be considered in future studies aiming at mitochondria-related phenomena like ageing and programmed cell death, complementarily to the well-established *P. anserina*-based and *S. cerevisiae*-based platforms^1^,^2^,^3^,^4^.

## Methods

### Culture media, construction of gene deletion and overexpression strains

For cultivation of *A. nidulans* strains, a standard complete medium containing 0.5% yeast extract^29^ and minimal nitrate medium (MNM) were used with appropriate nutritional supplements^54^.

Target genes in the *A. nidulans* genome were identified and confirmed by based on annotation data in the Broad Institute *Aspergillus* Comparative Database as well as by using *S. cerevisiae* and *P. anserina* homologues in protein *vs.* protein queries in NCBI BLAST (blastp; http://blast.ncbi.nlm.nih.gov/Blast.cgi? PROGRAM=blastp&PAGE_TYPE=BlastSearch&LINK_LOC=blasthome) and in the *P. anserina* Genome Project website (blastp; http://podospora.igmors.u-psud.fr/blast.php). The selected target genes with locus IDs and homologies to relevant *P. anserina* genes were *aodA* (encoding mitochondrial alternative oxidase, AN2099.2^25^, Expect value: 4e-123 when compared to *P. anserina Pa_3_1710* coding for alternative oxidase, which is identical to *PaAox*^6^), *dnmA* (hypothetical protein gene, AN8874.2, Expect value: 0.0 to *Pa_1_12670* coding for dynamin-related protein 1 DNM1 involved in mitochondrial fission, which is identical to *PaDnm1*^18^), *mnSOD* (putative manganese superoxide dismutase gene, AN5577.2, Expect value: 1E-102 to *P. anserina Pa_5_1740* encoding superoxide dismutase, which is identical to *PdSod3*^55^) and *pimA* (hypotetical protein gene, AN6193.2, Expect value: 0.0 to *Pa_3_4170* encoding putative Lon protease, mitochondrial precursor, which is identical to *PaLON*^15^). To construct deletion mutants, the double-joint PCR (DJ-PCR) method of Yu *et al.*^56^ was used with primers listed in Supplementary Table S2. The amplified deletion cassettes were used to transform RJMP1.59 strain using the Vinoflow FCE lysing enzyme (Novo Nordisk)^57^. Single copy transformants were selected after Southern blot analysis and crossed with rRAW16 to get prototroph strains. All progenies of the independent crosses were proved to be single copy deleted mutants by Southern analyses^56^. The genotypes of the studied *A. nidulans* strains are summarized in Supplementary Table S3.

To generate overexpression (OE) mutants, individual ORFs were amplified with the primers presented in Supplementary Table S2. The amplicons were digested with restriction enzymes as indicated in Supplementary Table S2, and ligated between the *niiA* promoter and the trpC terminator in pHS11 (Park and Yu, unpublished data). The final plasmid containing ¾ *pyroA* gene was expected to have single copy integration at the *pyroA* locus, which together with the overexpression of each gene was confirmed by PCR and Northern blot analyses, respectively^58^.

In all assays, the prototrophic THS30.3 strain (for genotype see Supplementary Table S3) was used as the control strain.

### Stress sensitivity assays, apoptotic cell death of mutants, conidiospore heat stress-sensitivity and viability tests

To determine the stress sensitivity of mutants, the agar plate assays of Hagiwara *et al.*^32^ were used with slight modifications. Freshly grown (6 days) conidia (10^5^ suspended in 5 μl aliquots of physiological saline −0.01% Tween 80) were spotted on MNM agar plates in Petri dishes^54^ containing the following stress generating agents: oxidative stress: 6.0 mM H_2_O_2_, 0.08 mM menadione sodium bisulphite (MSB), 2.0 mM diamide, 0.08 mM *t*BOOH, osmotic stress: 1.5 M KCl, 1.5 M NaCl, 2.0 M sorbitol, cell wall stress: 75 μg/ml CongoRed, heavy metal stress: 300 μM CdCl_2_. Stress plates were incubated for 5 days at 37 °C^24^.

The small molecular mass antifungal protein produced by *Penicillium chrysogenum* (PAF) causes apoptosis-like cell death in sensitive filamentous fungi like *A. nidulans*^26^. In these assays, PAF supplemented MNM agar prepared in 12-well tissue culture plates were point-inoculated with 2 × 10^3^ conidia, and were incubated at 37 °C for 72 h^26^.

### Determination of vitality and physiological parameters in submerged cultures of *A. nidulans*

*A. nidulans* strains were pre-grown in Erlenmeyer flasks (500 ml) containing 100 ml complete medium (pH 6.5), where culture media were inoculated always with 5 × 10^7^ spores and incubated for 16 h at 37 °C and at 3.7 Hz shaking frequency. Following that, mycelia were collected by filtration on sintered glass, washed with sterile distilled water and transferred into MNM (100 ml aliquots) containing either 2% or no glucose, and were incubated at 37 °C and 3.7 Hz shaking frequency for 4, 10 (with extra glucose) or 24 h (without any extra glucose). Growth of the mutants was characterized by increases in the dry cell mass (DCM). To determine the vitalities of the carbon-starving cultures of the tested strains, the cultures were washed to minimal medium containing 1% glucose and increases in the dry cell mass (DCM) were recorded^24^.

The intracellular reactive species (RS) levels were characterized by the formation of 2′,7′-dichlorofluorescein (DCF) from 2′,7′-dichlorofluorescin diacetate. RS includes all reactive oxygen species (ROS) and all reactive nitrogen species, which are able to oxidize 2′,7′-dichlorofluorescin to DCF^24^,^27^. At the incubation times tested, 10 μmol/ml 2′,7′-dichlorofluorescin diacetate was added to 20 ml aliquots of the cultures, and after incubating further for 1 h in 100 ml culture flasks, the mycelia were harvested by centrifugation. The production of DCF was determined spectrofluorimetrically^24^,^59^.

Changes in the specific activities of certain antioxidant enzymes were also recorded in separate experiments. In these cases, cell-free extracts were prepared by X-pressing and centrifugation^59^. Specific catalase, glutathione peroxidase (GPx), glutathione reductase (GR), superoxide dismutase (SOD) activities were measured as before^59^.

Respiration was measured in an Oxigraph O_2_ electrode (Hansatech, UK) at 37 °C. The cytochrome c-dependent pathway and alternative oxidase respiration was inhibited with 1.0 mM KCN and 0.25 mM SHAM, respectively^29^.

Proteinase activities of carbon-starving (24 h starvation) cultures were measured using azocasein as substrate^30^.

Prior to secondary metabolite analysis, mycelial mass collected from submerged cultures were lyophilized and resuspended in 70% v/v acetone (15 mg freeze-dried mycelium in 200 μl solvent)^24^. Following that, secondary metabolites in 25 μl aliquots of extracts were analyzed on silica gel TLC plates using sterigmatocystin (ST) standard.

### Morphological studies on mitochondria

Fungal conidia (10^3^ to 10^4^ ml^−1^) were grown in complete medium^30^ on coverslips for 20–24 h and washed in MNM before loading with 30 nM MitoTracker Green for 30 min, 2.5 nM dihydroethidium for 20 min and 2.5 nM Calcofluor White (CFW) for 5 min to visualize mitochondria, intracellular superoxide radicals and chitin component of the cell wall, respectively. Then the coverslips were mounted on the microscope chamber and images were obtained using a laser scanning microscope LSM 510 Meta (Zeiss, Jena, Germany)^23^,^31^.

Three-channel confocal Z-stack images were taken where the green channel showed mitochondria stained by MitoTracker Green, the red channel visualized the superoxide indicator dihydroethidium, while the blue channel (Calcofluor White staining) showed structure of the hypha and the boundaries for each segment (Fig. 3A).

In order to remove high-frequency noise, 2D stationary wavelet transform (SWT) based denoising was performed^60^ with delta parameter of 0.7. A region of interest (ROI) was manually marked on the Z-frame showing most of the hypha in focus respecting the boundaries of a single hyphal segment (Fig. 3B–f; dashed yellow line). A cell-free background (Fig. 3B–f; dashed white line) was also selected and the average intensity in the region was subtracted from all intensity values on the frame. We selected the region between the first and second septa behind the tip. Since the dye used for the green channel stained mitochondria, every pixel within the ROI either belonged to a mitochondrion (high intensity pixels) or not (low intensity pixels). Segmentation was done on the W2 level of the 2D SWT of the analyzed frame (Fig. 3B–e) using a noise level related threshold value optimized for every record. Actual threshold values varied between 0.08–0.20 and pixels above this threshold were marked as mitochondria (Fig. 3B–g). The volumetric ratio of mitochondria within the hyphal segment is given as the number of high intensity pixels compared to the total number of pixels within the selected ROI. Contagious areas of high intensity pixels were marked as mitochondria (Fig. 3B–f; solid white lines) and their sizes were calculated based on the number of pixels. The average of three consecutive Z-stacks was taken into account. The diameter of the hypha was measured in the blue channel (Calcofluor White staining) by the LSM Image Browser (Zeiss, Jena, Germany) program using the segments where the mitochondrial structure was examined.

### Sexual and asexual developments

To induce cleistothecium formation, 6 d old conidia were plated in top agar at 1 × 10^5^ conidia/plate density and were incubated at 37 °C. After 24 h incubation, plates were sealed with Parafilm and samples were taken with a cork borer every day between 3–12 d of incubation, and cleistothecia/cm^2^ were counted under a dissection microscope and cleistothecia/cm^2^ values were calculated^46^.

The conidiospore forming capabilities of the *A. nidulans* strains were determined as published by Vargas-Pérez *et al.*^33^. Briefly, conidia (10^5^) of the mutant and control strains were spotted onto MNM agar plates as described above, and were incubated and were allowed to sporulate at 37 °C for 5 days. Conidia were harvested by washing, counted in a Burker chamber and spore numbers were expressed as number/cm^2^ of colony surface. The areas of the colony surfaces were calculated using photographs.

To test the heat sensitivity of asexual spores, conidia were harvested from 6 days old colonies and suspended in physiological saline−0.01% Tween 80^24^. Conidia in 10^5^/ml concentration were incubated at 50 °C for 10 min and, following that, were diluted and spread on MNM agar plates. The numbers of colonies representing successfully germinated conidia were counted after incubation for 2 days at 37 °C. Conidia without any heat treatment were used as reference. For viability test, conidia were stored at 4 °C and germination rates were determined after 3, 6 and 12 d storages as described above^32^.

### Statistical analysis of experimental data

All experiments were performed in three independent sets with the exception of the mitochondrial volumetric ratio and size determinations where four independent experiments were carried out. Mean±SD values are presented, statistical significances were calculated using Student’s t-test, and *p*-values less than 0.05 were considered as statistically significant.

## Additional Information

**How to cite this article**: Leiter, É. *et al.* Characterization of the *aodA*, *dnmA, mnSOD* and *pimA* genes in *Aspergillus nidulans*. *Sci. Rep.* **6**, 20523; doi: 10.1038/srep20523 (2016).

## Supplementary Material

Supplementary Information

## Figures and Tables

**Table 1:** Changes in the specific DCF production and in the specific GPx, GR, catalase and SOD activities^a^ in growing cultures.

Strains	DCF	GPx	GR	Catalase	SOD
Control (THS30.3)	0.76 ± 0.08	3.63 ± 0.06	4.2 ± 0.1	0.94 ± 0.02	0.082 ± 0.009
Δ*aodA*	3.40 ± 0.05***	2.6 ± 0.1***	4.6 ± 0.2*	2.3 ± 0.1***	0.14 ± 0.02**
*aodA*OE	0.9 ± 0.3	4.19 ± 0.06***	4.7 ± 0.3*	0.8 ± 0.1*	0.09 ± 0.01
Δ*dnmA*	0.8 ± 0.2	5.8 ± 0.2***	7.6 ± 0.3***	1.7 ± 0.1***	0.130 ± 0.009***
*dnmA*OE	0.59 ± 0.07*	2.78 ± 0.03***	4.0 ± 0.1	1.3 ± 0.1***	0.078 ± 0.006
Δ*mnSOD*	0.5 ± 0.1*	3.62 ± 0.03	4.6 ± 0.1*	1.2 ± 0.1*	0.063 ± 0.004*
*mnSOD*OE	0.6 ± 0.1*	3.6 ± 0.1	4.6 ± 0.2*	0.68 ± 0.03*	0.15 ± 0.03*
Δ*pimA*	1.8 ± 0.4**	4.7 ± 0.1***	4.7 ± 0.2**	1.49 ± 0.05***	0.088 ± 0.008
*pimA*OE	2.4 ± 0.3***	3.67 ± 0.06	4.6 ± 0.2*	1.1 ± 0.1	0.074 ± 0.003

^a^Specific enzyme activities are expressed as mkat/kg protein (GPx and GR) or kat/kg protein (catalase). Specific SOD activities are given as mU/kg protein^59^. Specific DCF productions are expressed as mmol/kg DCM. Specific DCF productions as well as specific enzyme activity values are presented as mean ± SD calculated from three independent experiments. *p < 5%, **p < 1% and ***p < 0.1%. *p* values were calculated using the Student’s t-test.

**Table 2:** Changes in the specific DCF and ST productions and in the specific GPx, GR, catalase and SOD activities^a^ in carbon-starving ageing cultures.

Strains	DCF	ST production	GPx	GR	Catalase	SOD
Control (THS30.3)	0.18 ± 0.02	0.14 ± 0.02	0.62 ± 0.07	2.3 ± 0.2	2.9 ± 0.1	0.209 ± 0.009
Δ*aodA*	0.10 ± 0.01***	0.030 ± 0.004***	0.71 ± 0.01	2.39 ± 0.04	1.6 ± 0.2***	0.167 ± 0.009***
*aodA*OE	0.18 ± 0.01	0.026 ± 0.003***	0.33 ± 0.06***	1.3 ± 0.1***	1.4 ± 0.2***	0.10 ± 0.01***
Δ*dnmA*	0.28 ± 0.04*	0.024 ± 0.003***	0.57 ± 0.07	2.1 ± 0.1	3.2 ± 0.1	0.20 ± 0.01
*dnmA*OE	0.12 ± 0.01**	0.15 ± 0.02	0.36 ± 0.04***	1.67 ± 0.04***	1.8 ± 0.1***	0.13 ± 0.01***
Δ*mnSOD*	0.15 ± 0.01	0.18 ± 0.02*	0.59 ± 0.01	2.38 ± 0.06	2.7 ± 0.1	0.132 ± 0.005***
*mnSOD*OE	0.10 ± 0.01***	0.2 ± 0.02**	0.41 ± 0.01***	2.0 ± 0.1	1.0 ± 0.2***	0.138 ± 0.008***
Δ*pimA*	0.6 ± 0.1***	0.020 ± 0.002***	0.59 ± 0.01	1.9 ± 0.4	4.7 ± 0.2***	0.24 ± 0.03
*pimA*OE	0.27 ± 0.01***	0.35 ± 0.04***	0.24 ± 0.03***	1.03 ± 0.01***	1.4 ± 0.1***	0.12 ± 0.012***

^a^Specific enzyme activities are expressed as mkat/kg protein (GPx and GR) or kat/kg protein (catalase). Specific SOD activities are given as mU/kg protein^59^. Specific DCF and ST productions are expressed as mmol/kg DCM and mg/g DCM, respectively. Specific DCF and ST productions as well as specific enzyme activity values are presented as mean ± SD calculated from three independent experiments. *p < 5%, **p < 1% and ***p < 0.1%. *p* values were calculated using the Student’s t-test.

**Table 3:**
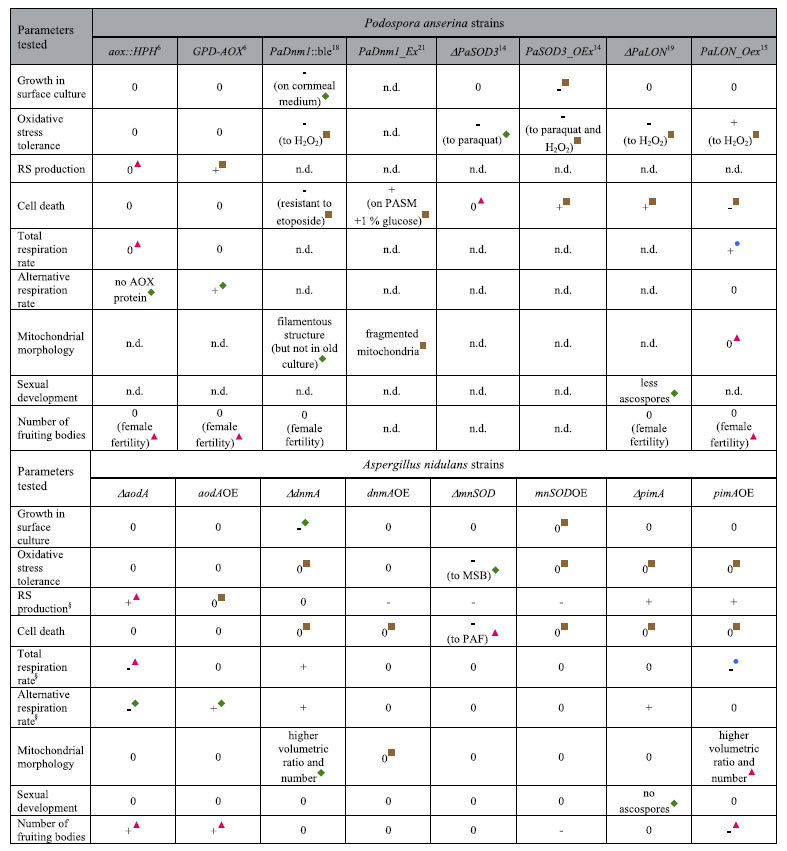
Comparison of the phenotypes of relevant *Podospora anserina* and *Aspergillus nidulans* mutant strains.

^§^Reactive species (RS) productions^24^,^27^ and respiration rates^29^ measured in growing cultures were taken into consideration.

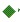
The same or similar phenotypes were described in the relevant *P. anserina* and the *A. nidulans* mutants.

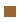
Phenotype was observed in *P. anserina* but not in the appropriate *A. nidulans* mutant.

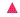
Phenotype was observed in *A. nidulans* but not in the appropriate *P. anserina* mutant.

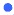
Opposite phenotypes in the relevant *P. anserina* and the *A. nidulans* mutants.

0 stands for no alteration meanwhile + and – indicate positive or negative alterations in comparison to appropriate controls; n.d.-not determined.
