# Supplementary material for: Characterization of the aodA, dnmA, mnSOD and pimA genes in Aspergillus nidulans
Source: Sci Rep. 2016 Feb 5;6:20523. doi: 10.1038/srep20523 (PMC4742808; doi:10.1038/srep20523)
Supplement: Supplementary Information [file srep20523-s1.pdf]

Characterization of the *aodA, dnmA, mnSOD* and *pimA* genes in *Aspergillus nidulans*

Éva Leiter, Hee-Soo Park, Nak-Jung Kwon, Kap-Hoon Han, Tamás Emri, Viktor Oláh, Ilona Mészáros, Beatrix Dienes, János Vincze, László Csernoch, Jae-Hyuk Yu & István Pócsi

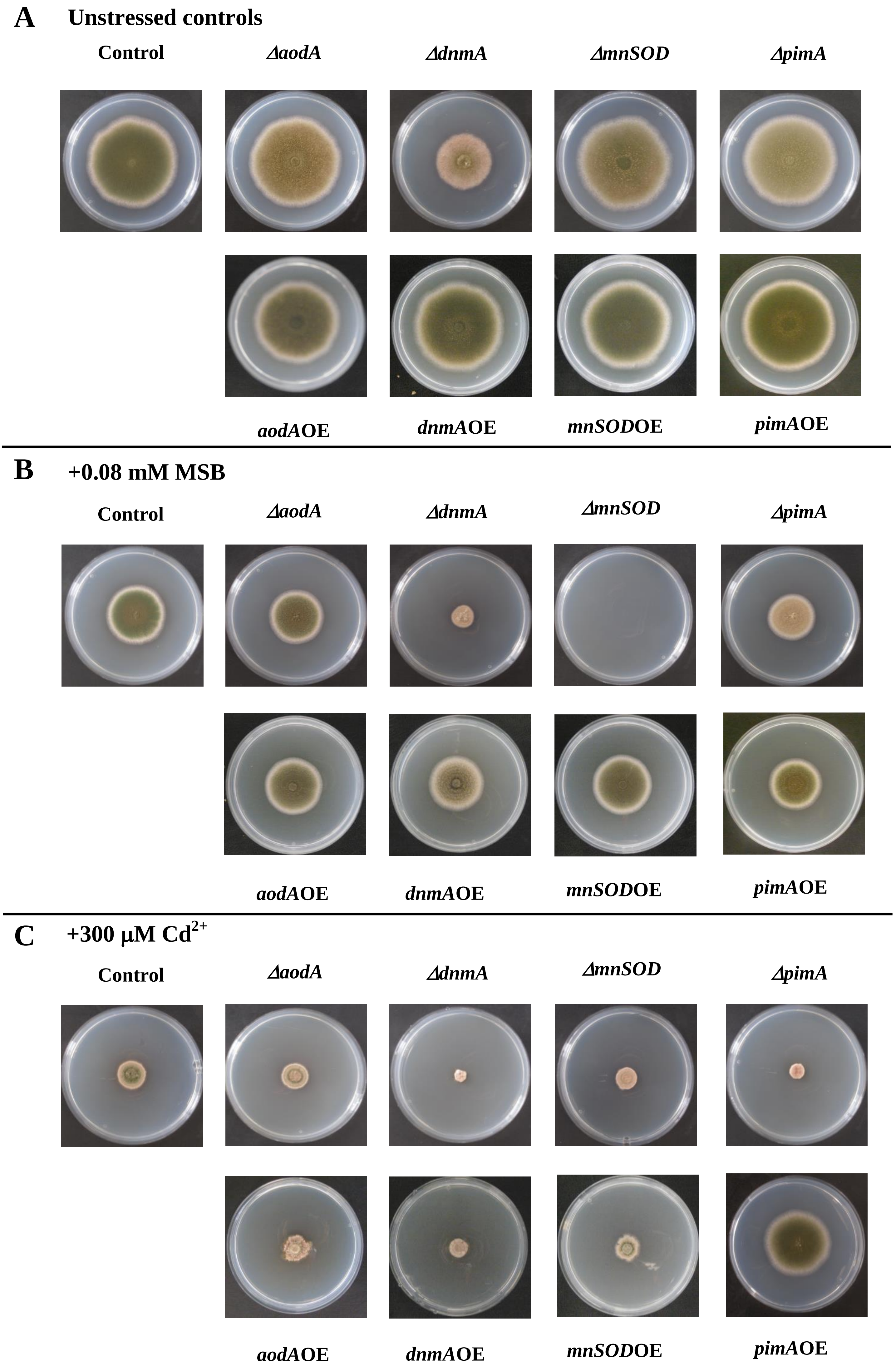
A
Unstressed controls
Control
ΔaodA
ΔdnmA
ΔmnSOD
ΔpimA
aodAOE
dnmAOE
mnSODOE
pimAOE
B
+0.08 mM MSB
Control
ΔaodA
ΔdnmA
ΔmnSOD
ΔpimA
aodAOE
dnmAOE
mnSODOE
pimAOE
C
+300 μM Cd^{2+}
Control
ΔaodA
ΔdnmA
ΔmnSOD
ΔpimA
aodAOE
dnmAOE
mnSODOE
pimAOE

D

Control | ***ΔaodA*** | ***ΔdnmA*** | ***ΔmnSOD*** | ***ΔpimA***

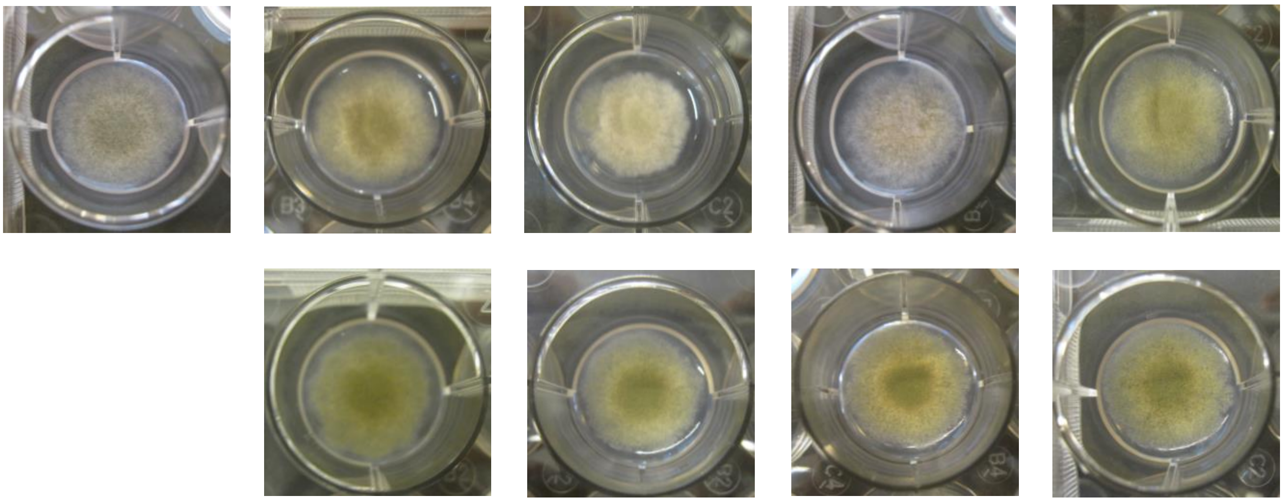

***aodA*OE** | ***dnmA*OE** | ***mnSOD*OE** | ***pimA*OE**

F **+200 μg/ml PAF**

Control | ***ΔaodA*** | ***ΔdnmA*** | ***ΔmnSOD*** | ***ΔpimA***

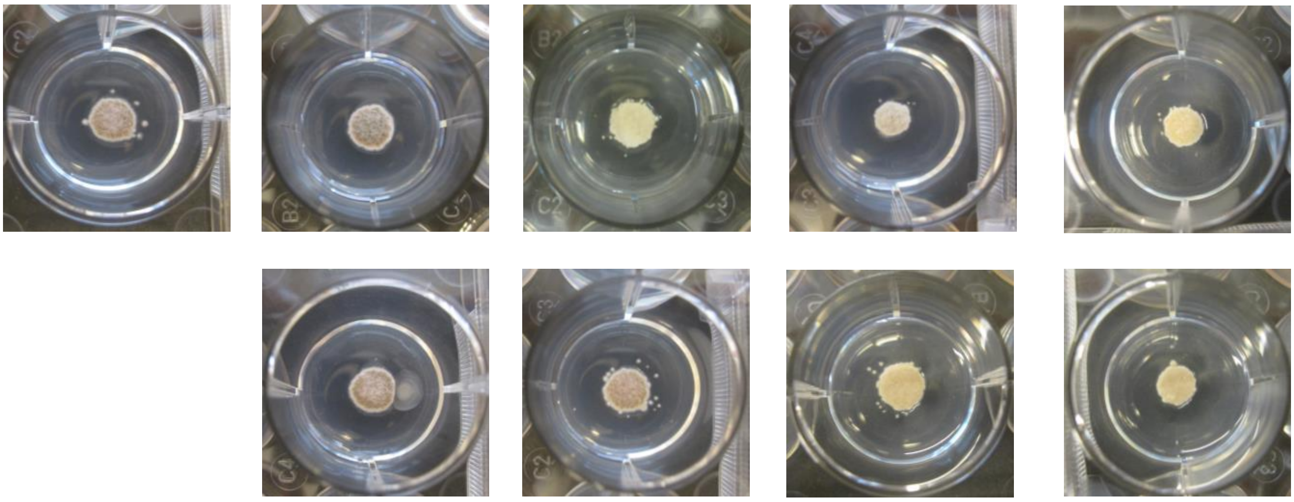

***aodA*OE** | ***dnmA*OE** | ***mnSOD*OE** | ***pimA*OE**

Figure S1. Comparison of the growth and the stress sensitivities of the control and mutant strains in surface cultures. Part A: Growth of control (THS30.3) and mutant strains on MNM agar plates in Petri dishes. Part B: Stress sensitivities recorded in the presence of 0.08 mM MSB. Part C: Growth inhibitions observed in MNM agar plates supplemented with 300 μM $CdCl_2$. Part D: Growth of control and mutant strains on MNM agar in 12-well tissue culture plates. Part E: PAF (200 μg) sensitivities of tested strains. Agar plates in Petri dishes shown in Parts A-C were point-inoculated with 10^5 conidia and were incubated at 37 °C for 5 days[24]. In Parts D and E, MNM agar prepared in 12-well tissue culture plates were point-inoculated with 2 x 10^3 conidia, and were incubated at 37 °C for 72 h[26]. For all mutant strains, per cent growth inhibition values were calculated and were compared to those determined for the control strain under each stress condition tested.

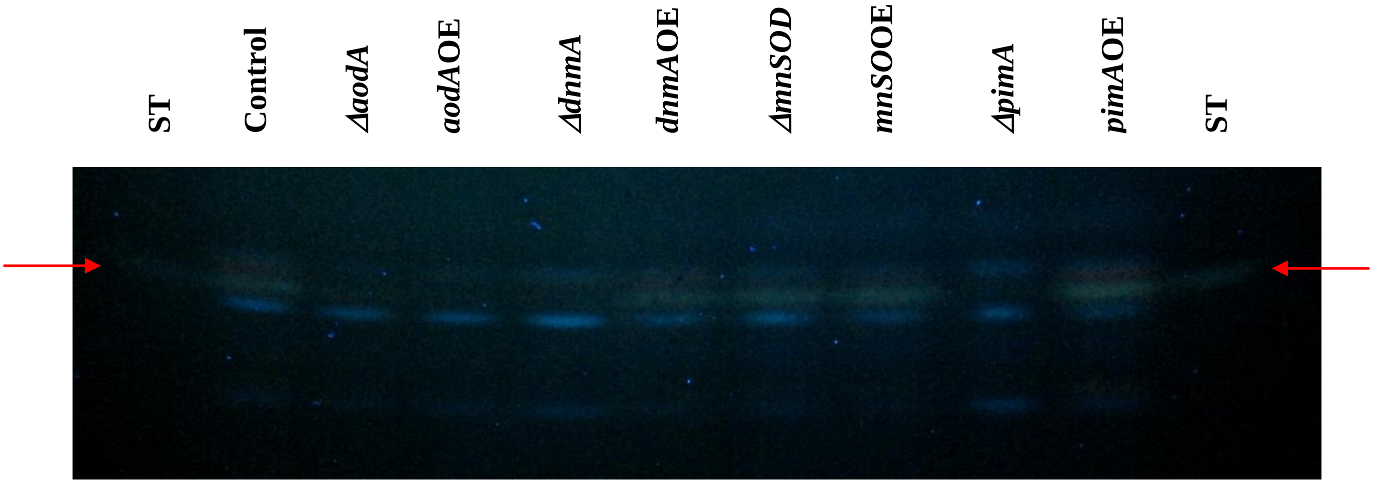

Figure S2. Secondary metabolite production by the control (THS30.3) and mutant strains. Typically, 25 μL aliquots of extracts of lyophilised mycelia (15 mg quantities were extracted in 200 μl aliquots of 70 % acetone) were spotted onto the silica gel plates[24,SR1]. TLC plates were developed in toluene/ethylacetate/glacial acetic acid=80:10:10, sprayed with 1% w/v $AlCl_3$ in ethanol and visualized under long-wave ultraviolet light. Sterigmatocystin (ST) bands were identified using a standard solution (0.14 μg ST in 25 μL 70 % acetone) and ST productions were quantified by videodensitometry[24,SR1]. Arrows indicate the migration position of ST.

Supplementary Reference to Figure S2

SR1. Tóth, V., Nagy, C.T., Miskei, M., Pócsi, I. & Emri, T. Polyphasic characterization of "*Aspergillus nidulans* var. *roseus*" ATCC 58397. *Folia Microbiol* **56,** 381-388 (2011).

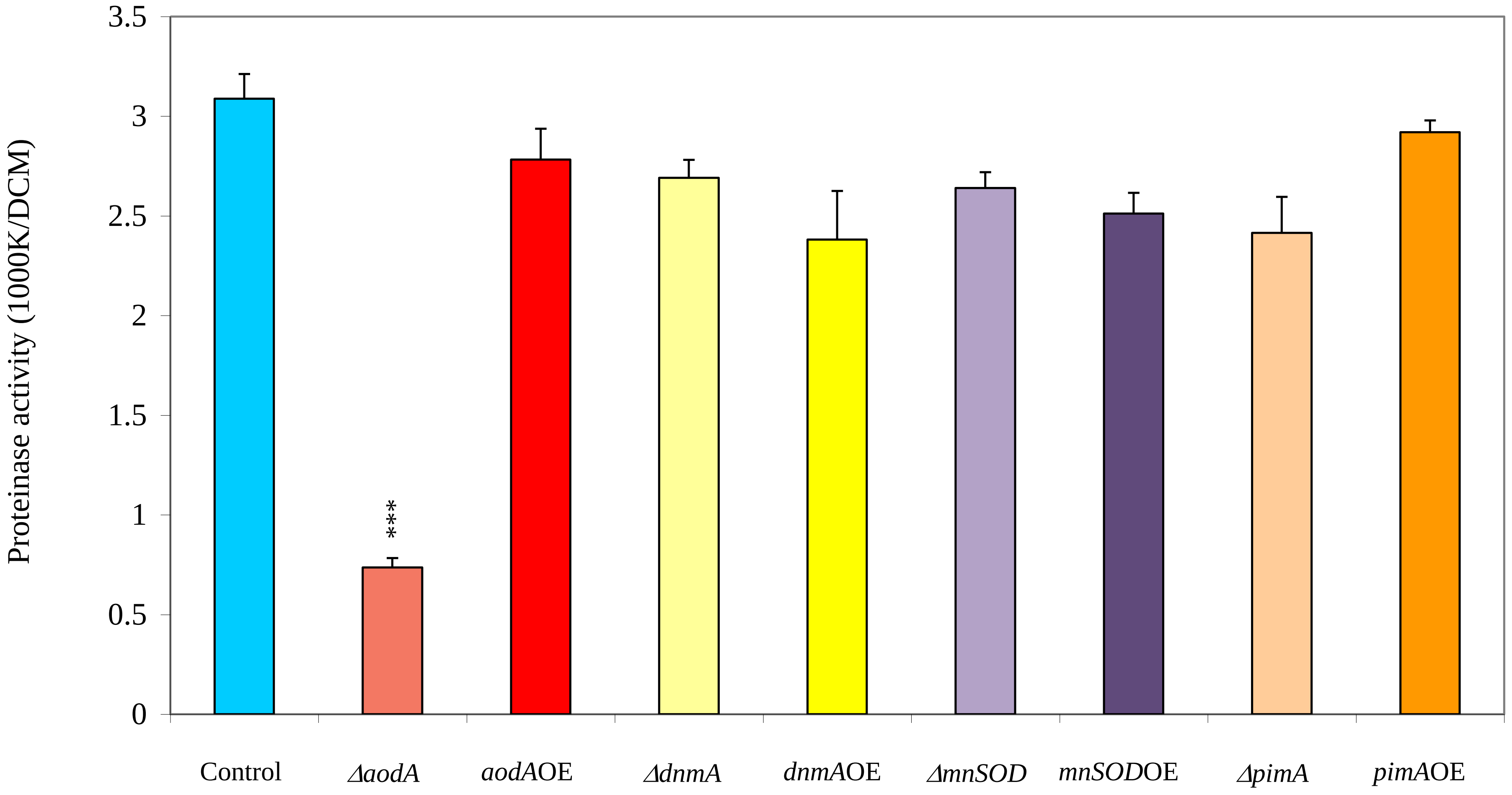
Proteinase activity (1000K/DCM)
3.5
3
2.5
2
1.5
1
0.5
0
***
Control
ΔaodA
aodAOE
ΔdnmA
dnmAOE
ΔmnSOD
mnSODOE
ΔpimA
pimAOE

Figure S3. Proteinase activities in carbon-depleted submerged cultures of the control (THS30.3) and mutant strains. Mean±SD values calculated from three independent experiments are presented. Statistically significant differences determined by the Student's t test are marked with asterisks: *** p<0.1 %.

A

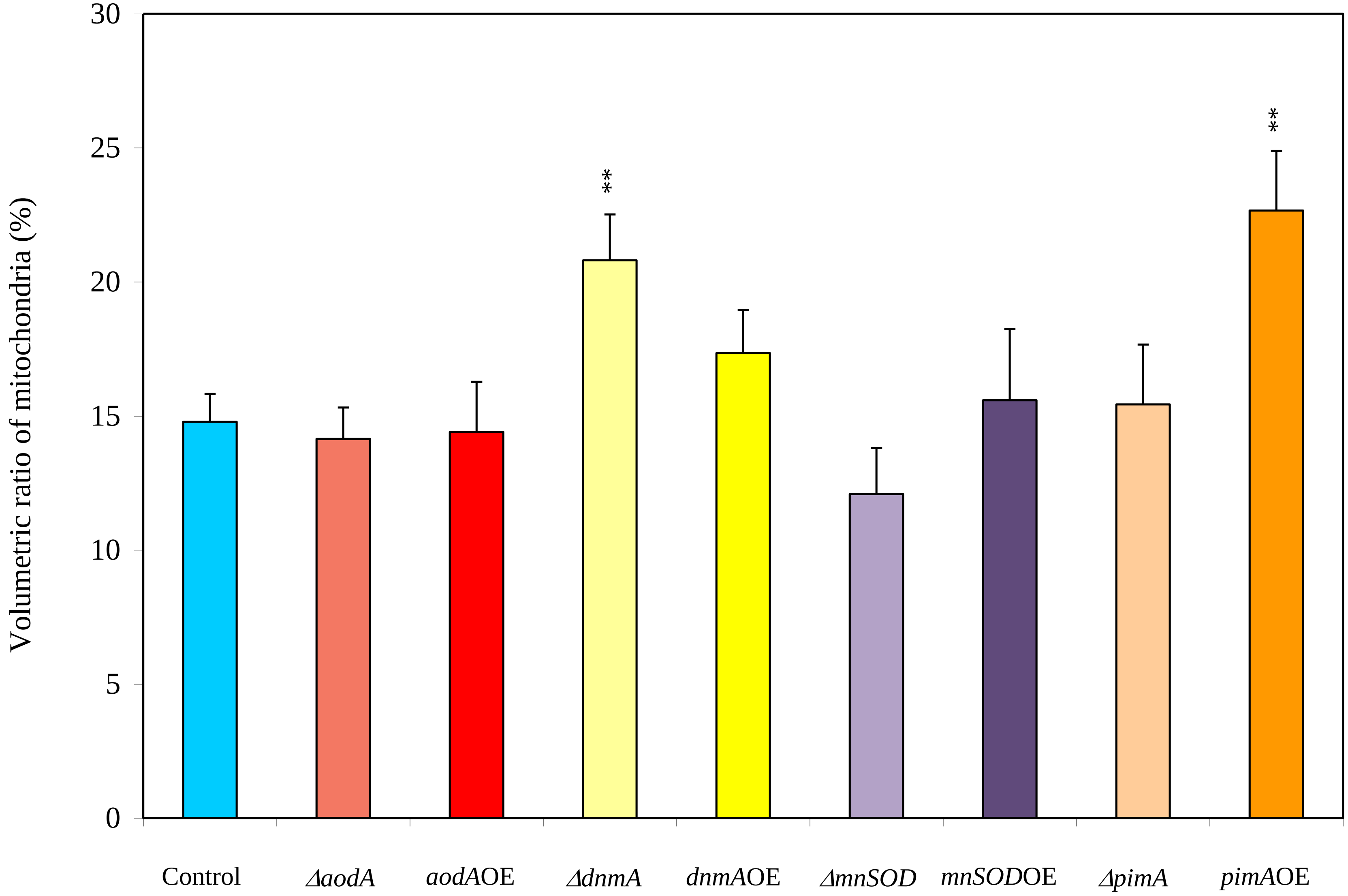

Volumetric ratio of mitochondria (%)
30
25
20
15
10
5
0
**
**
Control
ΔaodA
aodAOE
ΔdnmA
dnmAOE
ΔmnSOD
mnSODOE
ΔpimA
pimAOE

B

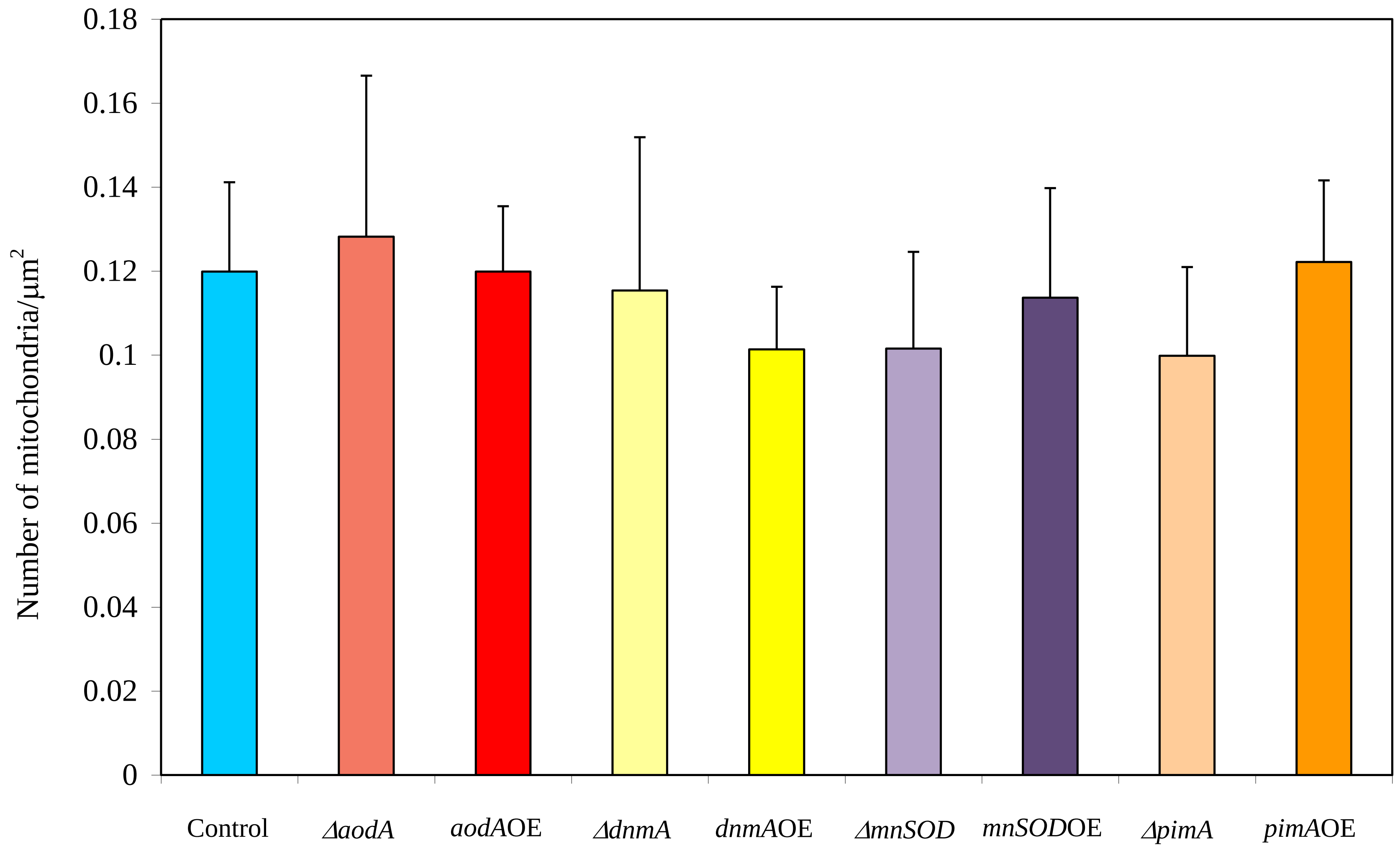
0.18
0.16
0.14
0.12
0.1
0.08
0.06
0.04
0.02
0
Number of mitochondria/μm^2
Control
ΔaodA
aodAOE
ΔdnmA
dnmAOE
ΔmnSOD
mnSODOE
ΔpimA
pimAOE

C

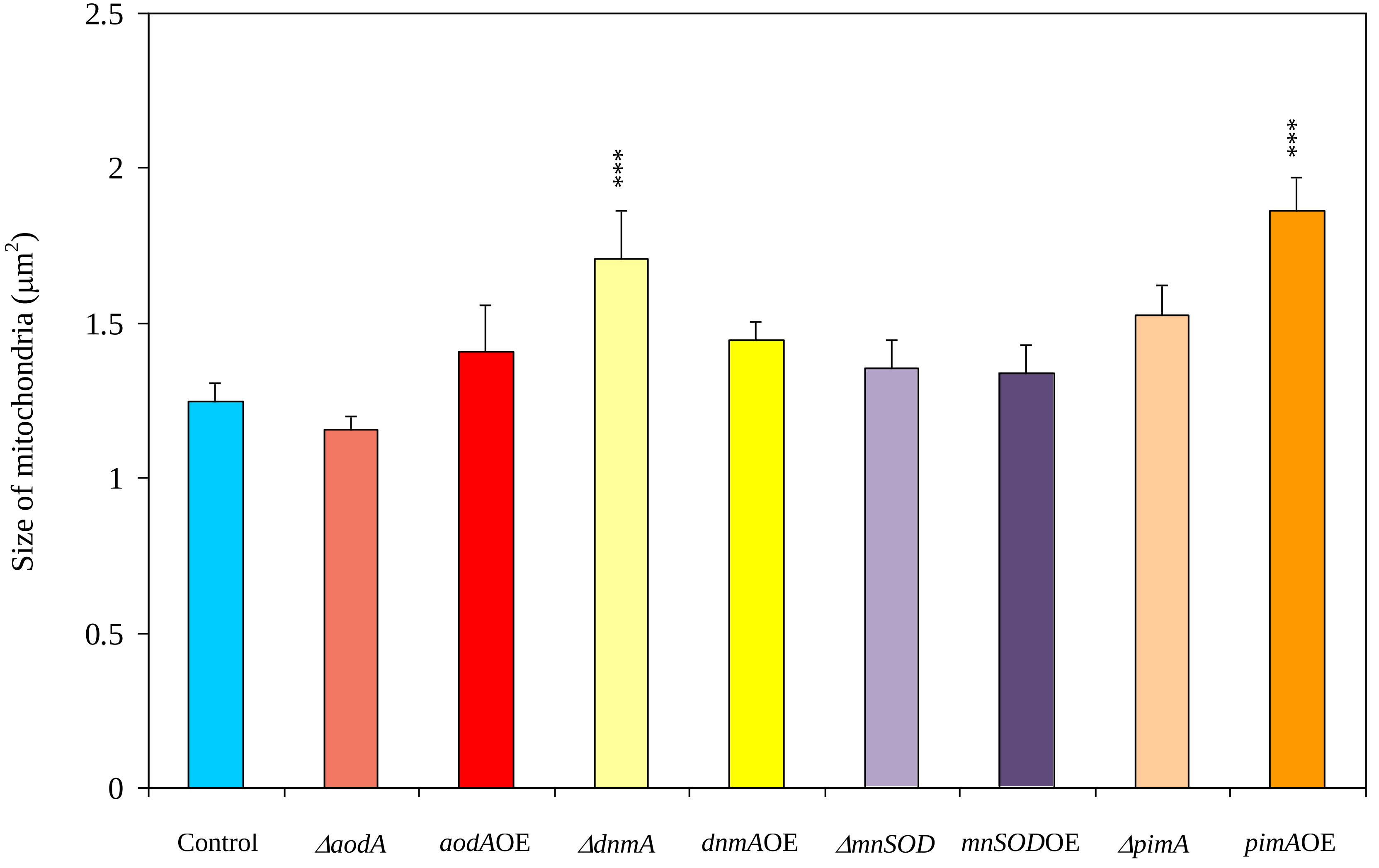

Size of mitochondria (μm²)
2.5
2
1.5
1
0.5
0
***
***
Control
ΔaodA
aodAOE
ΔdnmA
dnmAOE
ΔmnSOD
mnSODOE
ΔpimA
pimAOE

D

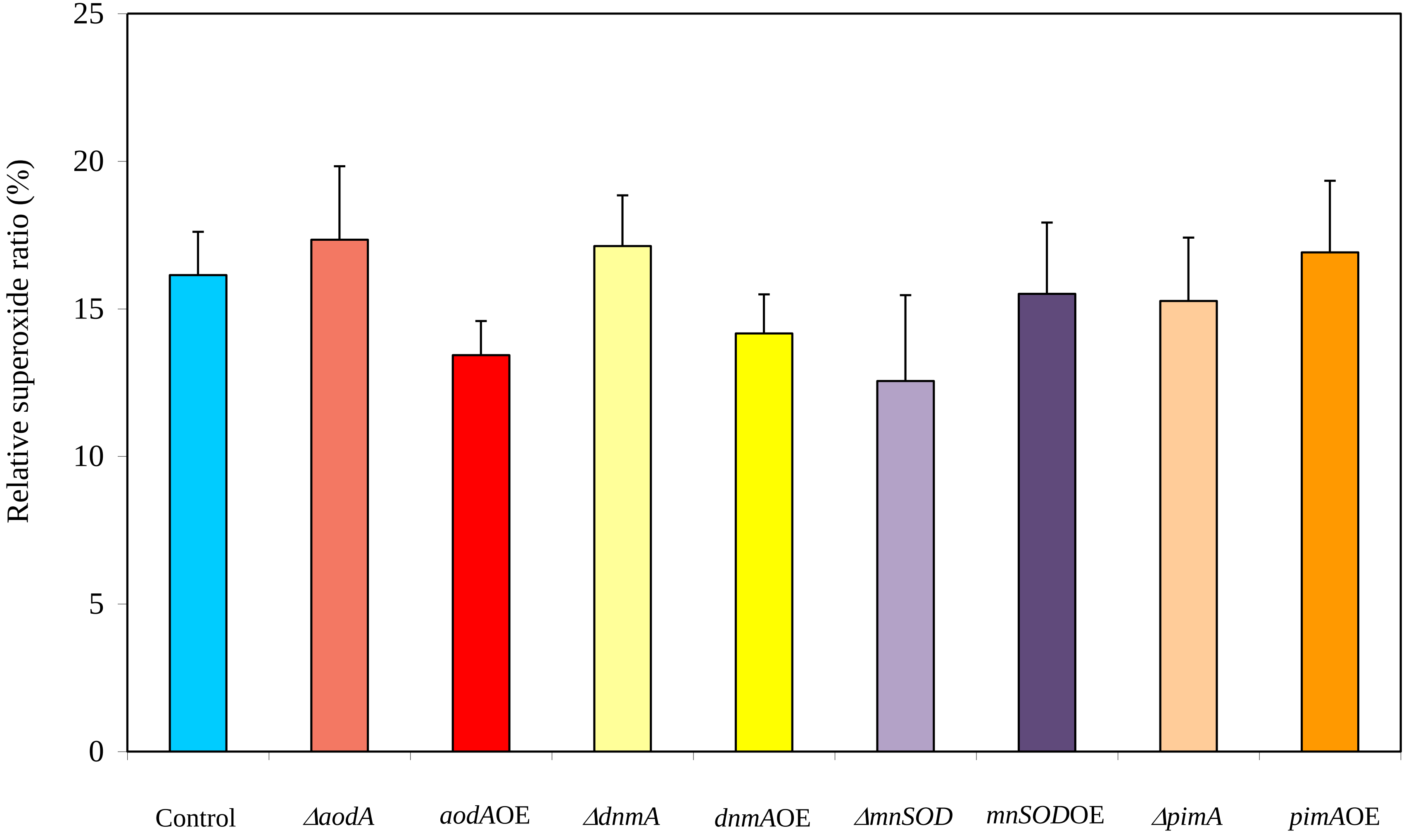

Relative superoxide ratio (%)
25
20
15
10
5
0
Control
ΔaodA
aodAOE
ΔdnmA
dnmAOE
ΔmnSOD
mnSODOE
ΔpimA
pimAOE

E

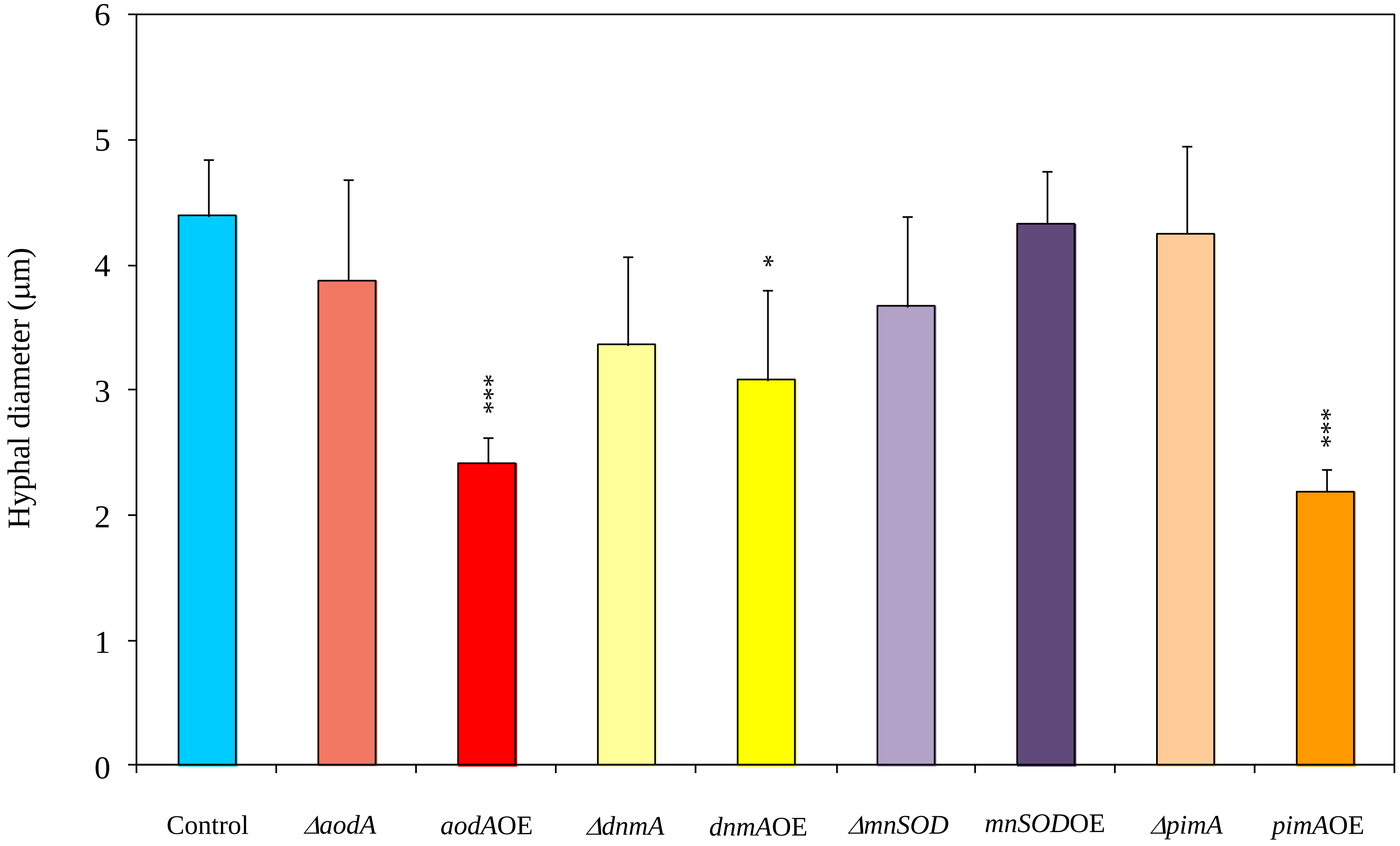

Hyphal diameter (µm)
6
5
4
3
2
1
0
***
*
***
Control
ΔaodA
aodAOE
ΔdnmA
dnmAOE
ΔmnSOD
mnSODOE
ΔpimA
pimAOE

Figure S4. Comparison of the volumetric ratio of mitochondria (Part A), the number (Part B) and size (Part C) of mitochondria, relative superoxide ratio (Part D) as well as the diameter of hyphae (Part E) in the second hyphal segment of the control (THS30.3) and mutant strains. Hyphal segments, mitochondria and superoxide ratio were visualized with Calcofluor White, Mitotracker Green and dihydroethidium staining, respectively[23,31,SR1]. Mean±SD values calculated from four independent experiments are presented. Statistically significant differences determined by the Student's t test are marked with asterisks: * p<5%, ** p<1%, *** p<0.1%

Supplementary Reference to Figure S4

SR1 Galgóczy, L. et al. Investigation of the antimicrobial effect of *Neosartorya fischeri* antifungal protein (NFAP) after heterologous expression in *Aspergillus nidulans*. *Microbiology-UK* **159,** 411-419 (2013).

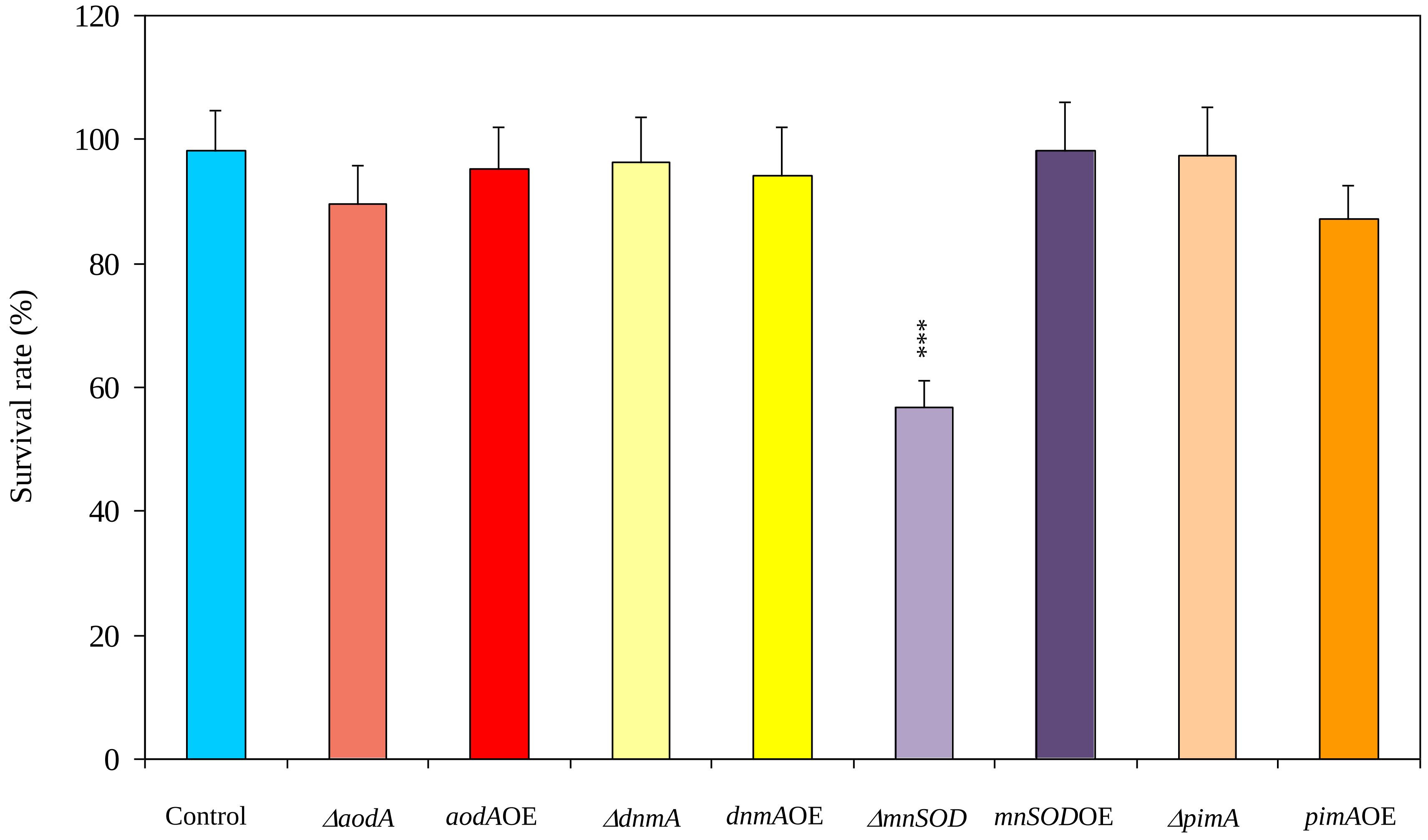

120
100
80
60
40
20
0
Survival rate (%)
***
Control
ΔaodA
aodAOE
ΔdnmA
dnmAOE
ΔmnSOD
mnSODOE
ΔpimA
pimAOE

Figure S5. Survival rates of conidiospores of the control (THS30.3) and the mutant strains after heat stress. Conidia in 10^5/ml concentration (in 1 ml) were incubated at 50 ^{o}C for 10 min and, following that, were diluted and spread on MNM agar plates. The numbers of colonies representing successfully germinated conidia were counted after incubation at 37 ^{o}C for 2 d. Conidia without any heat treatment were used as control[32]. Mean±SD values calculated from three independent experiments are presented. The statistically significant difference determined by the Student's t test is marked with asterisks: *** p<0.1%

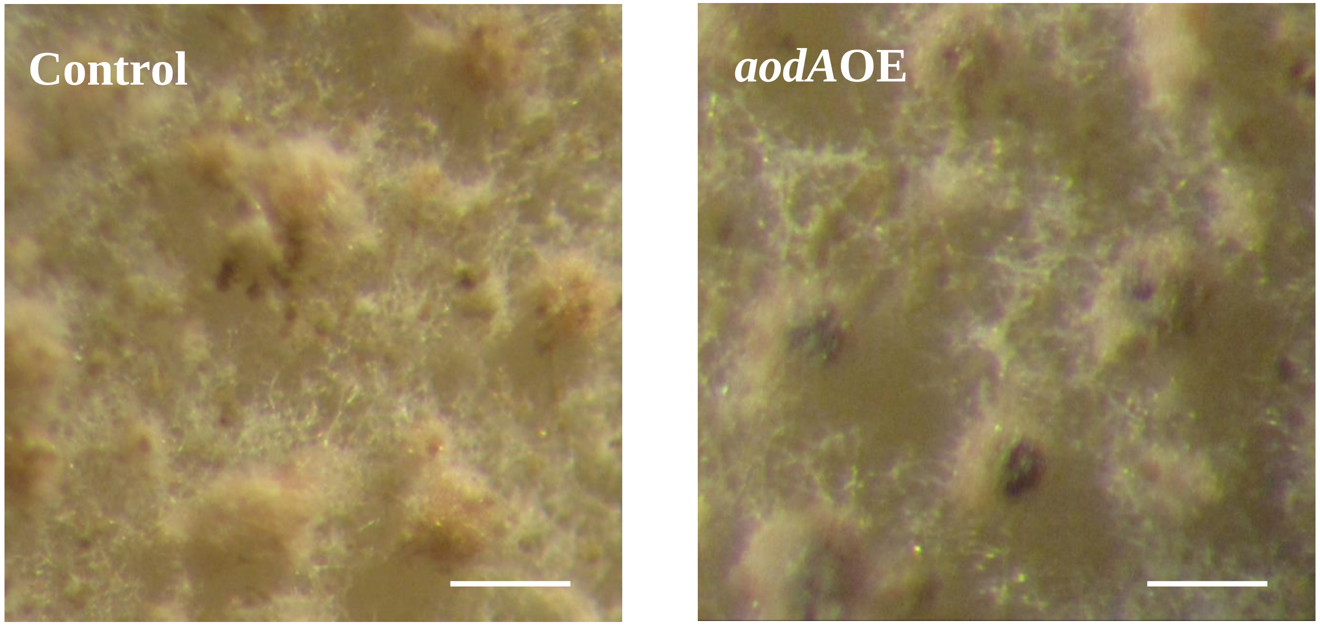

Figure S6. Cleistothecia of the the THS30.3 control and the *aodA*OE mutant strains. Conidia from selected strains were plated in top agar at $1x10^5$ conidia/plate density and incubated at 37 °C. After 24 h incubation, plates were sealed with Parafilm and samples were taken and cleistotechia formations were checked every day between 3-12 days of incubation. The numbers of cleistothecia were counted under a dissection microscope and were expressed as cleistothecia/cm^2. Photographs show 4 d cleistothecia and the bars equal to 200 µm.

Note S1. In Fig. 5, schematic representations of fissioning *A. nidulans* mitochondria, a hypha, a cleistothecium with asci (sexual fruiting body) and a conidiophore with conidia (asexual sporulation) are presented. Mitochondria (green) possess lamellae aligned parallel with the long axes of the organelles[SR1]. Although no 3D structure is available for the studied *A. nidulans* mitochondrial proteins, their hypothetical structures and shapes are based on homologous proteins and are adapted from several publications as follows: MnSOD: the homotetrameric MnSOD3 of *Candida albicans*[SR2,SR3], PimA: the ring-shaped Pim1 protease of *S. cerevisiae* incorporating seven subunits[SR4,SR5], AodA: the alternative oxidase of *Trypanosoma brucei*[SR6] and DnmA: the Dnm1 dynamin-related protein of *S. cerevisiae*[SR7]. The schematic hypha shows only the hyphal tip and the first subapical compartment between the first and second septa with nuclei (blue) and mitochondria (green). For a more detailed subcellular organization and dynamics of *A. nidulans* hyphae, you may consult several relevant papers[SR1,SR8]. The cleistothecium (blue) with sacs of ascospores (red) lies on a mat of hyphae and globose Hülle cells as adapted from the publication of Park et al.[SR9]. Asexual development results in the emergence of conidiophores with stalk and vesicle (white) and layers of metulae and phialide cells with conidia (blue). The picture was adapted from the paper of Casselton and Zolan[SR10], and to gain a more detailed view on asexual development, a number of review papers are available, *e.g.*[44,45,SR11].

Supplementary References to Note S1

SR1. Leiter, É. et al. Antifungal protein PAF severely affects the integrity of the plasma membrane of *Aspergillus nidulans* and induces an apoptosis-like phenotype. *Antimicrob Agents Chemother* **49,** 2445-2453 (2005).

SR2. Sheng, Y. et al. Comparison of two yeast MnSODs: mitochondrial *Saccharomyces cerevisiae* versus cytosolic *Candida albicans*. *J Am Chem Soc* **133,** 20878-20889 (2011).

SR3. Sheng, Y. et al. Crystal structure of cytosolic MnSOD3 from *Candida albicans*. Biological assembly image for 3QVN. *RCSB Protein Data Bank*, http://www.rcsb.org/pdb/explore.do?structureId=3QVN (2011).

SR4. Stahlberg H. et al. Mitochondrial Lon of *Saccharomyces cerevisiae* is a ring-shaped protease with seven flexible subunits. *Proc Natl Acad Sci USA* **96**, 6787-6790 (1999).

SR5. Van Dyck, L. and Langer, T. ATP-dependent proteases controlling mitochondrial function in the yeast *Saccharomyces cerevisiae*. *Cell Mol Life Sci* **56,** 825-842 (1999).

SR6. Shiba, T. et al. Structure of the trypanosome cyanide-insensitive alternative oxidase. *Proc Natl Acad Sci. USA* **110**, 4580-4585 (2013).
SR7. Mears, J.A. et al. Conformational changes in Dnm1 support a contractile mechanism for mitochondrial fission. *Nat Struct Mol Biol* **18**, 20-26 (2011).
SR8. Peñalva, M.A. Tracing the endocytic pathway of *Aspergillus nidulans* with FM4-64. *Fungal Genet Biol* **42,** 963-975 (2005).
SR9. Park, H.S., Nam, T.Y., Han, K.H., Kim, S.C. & Yu, J.H. VelC positively controls sexual development in *Aspergillus nidulans*. *PLoS One* **9,** e89883 (2014).
SR10. Casselton, L. & Zolan, M. The art and design of genetic screens: filamentous fungi. *Nat Rev Genet* **3,** 683-697 (2002).
SR11. Adams, T.H., Wieser, J.K. & Yu, J.H. Asexual sporulation in *Aspergillus nidulans*. *Microbiol Mol Biol Rev* **62,** 35-54 (1998).

Table S1. Growth of *A. nidulans* strains recorded in glucose supplemented and glucose-starved cultures

Strains	Experimental arrangements (glucose supplementation)					
	Supplemented with 2 % glucose at 16 h incubation.			No glucose supplementation		+ 1 % glucose added after 16 + 24 h incubation
	16 h	*16+4 h*	*16+10 h*	*16 h*	*16+24 h*	*16+24+10 h*
	Biomass (DCM) productions (g/l)[1]					
Control (THS30.3)	2.8±0.3	7.3±0.6	7.6±0.6	2.6±0.3	1.9±0.1	4.9±0.4
ΔaodA	2.8±0.3	3.8±0.4***	7.2±0.8	2.7±0.3	2.6±0.3**	5.4±0.4
*aodA*OE	2.7±0.2	6.3±0.6	6.9±0.6	2.7±0.2	2.2±0.2	5.0±0.4
ΔdnmA	2.3±0.2	5.7±0.6*	7.3±0.8	2.4±0.3	2.0±0.1	4.0±0.3
*dnmA*OE	2.5±0.2	4.0±0.4***	7.7±0.7	2.4±0.2	2.3±0.3	5.0±0.4
ΔmnSOD	2.4±0.3	6.3±0.6	7.3±0.8	2.7±0.3	2.2±0.2	4.8±0.4
*mnSOD*OE	2.5±0.3	4.7±0.5**	6.5±0.6	2.7±0.3	2.0±0.2	4.7±0.4
ΔpimA	2.6±0.3	5.5±0.7*	6.6±0.8	2.7±0.3	2.0±0.2	4.4±0.3
*pimA*OE	2.6±0.3	6.3±0.6	6.8±0.6	2.7±0.2	1.8±0.1	5.4±0.4

[1] - Mean±SD values calculated from three independent experiments are presented. Statistically significant differences between control and mutant strains determined by the Student's t test are marked with asterisks: * p<5 %, ** p<1 %, *** p<0.1 %

Table S2. Oligonucleotides used in this study.

Name	Sequence (5'→3')	Purpose
oEL-1	TCAGATTGCGTTCAAAGTCCATGG	5'-flanking region of *aodA*
oEL-2	GCTTTGGCCTGTATCATGACTTCA TGAGGCAGGAATCGGAACTCTCAG	5' *aodA* with *AfupyrG* tail
oEL-3	ATCGACCGAACCTAGGTAGGGTA TGGTTGGACTTGAGCATAGCATCG	3' *aodA* with *AfupyrG* tail
oEL-4	TGGTGTGAGCAGCAATTGTGGTGTG	3'-flanking region of *aodA*
oEL-5	TGTTTAACAAGTACCTCTACAGGTG	5' nested of *aodA*
oEL-6	ACCCTTCCCATCCAGCAGACCTG	3' nested of *aodA*
oEL-7	TGCTGGTAGAGCTATCAGGATTAG	5'-flanking region of *dnmA*
oEL-8	GCTTTGGCCTGTATCATGACTTCA ATGCAACCGTTCACAATCTTCTGG	5' *dnmA* with *AfupyrG* tail
oEL-9	ATCGACCGAACCTAGGTAGGGTA TCTAGCTCAAGTGGCGTGTTTACC	3' *dnmA* with *AfupyrG* tail
oEL-10	TCGCTTCAATTGCTGTTGGTTATC	3'-flanking region of *dnmA*
oEL-11	ATCCTTACCTGGTTAACAGGCTG	5' nested of *dnmA*
oEL-12	ATTCTATCAGTCGCGCTGCTTAC	3' nested of *dnmA*
oEL-13	AAGCGACTCCAACTGGTCAGTCAC	5'-flanking region of *mnSOD*
oEL-14	GCTTTGGCCTGTATCATGACTTCA TCCAGACACTGGCCACAGCTATAC	5' *mnSOD* with *AfupyrG* tail
oEL-15	ATCGACCGAACCTAGGTAGGGTA TTCCATGCAGTCATCCTGTAGTAG	3' *mnSOD* with *AfupyrG* tail
oEL-16	TGCAGGGATTCGTGGCCTTCTTG	3'-flanking region of *mnSOD*
oEL-17	TGACTTCAAGAGCTCCAAAGGCAG	5' nested of *mnSOD*
oEL-18	AGACCATCGGCATCGCTGTCGGTC	3' nested of *mnSOD*
oEL-19	AGCATCACATGTAGCAGCTACCTG	5'-flanking region of *pimA*
oEL-20	GCTTTGGCCTGTATCATGACTTCA AGAATATCACAATAGTCCCTGTCC	5' *pimA* with *AfupyrG* tail
oEL-21	ATCGACCGAACCTAGGTAGGGTA TGCGAATGCATGGTTCGGAGTTGC	3' *pimA* with *AfupyrG* tail
oEL-22	TGAGCCCAGAAATTCTGGCAGCTAG	3'-flanking region of *pimA*
oEL-23	TCAATGGAGTGATAACGATGTCTTG	5' nested of *pimA*
oEL-24	ACAGTAGTGTGCTTGATGTCCAAC	3' nested of *pimA*
oJH-83	GGATGTATCGTGACTGGCCTTCGG	5' *AfupyrG* marker
oJH-86	TAATTCGCGGCATACGGTGTCTAA	3' *AfupyrG* marker
oEL-25	GGAAGCTTATGAATTCGATGTCA	5' *aoxA* with *HindIII*
oEL-26	GGGCGGCCGCTCAAACAACCTCCTC	3' *aoxA* with *NotI*
oEL-27	GGAAGCTTATGGCCGCCTTGGGG	5' *dnmA* with *HindIII*
oEL-28	GGGCGGCCGCTTACAAAACTTCGCC	3' *dnmA* with *NotI*
oEL-29	GGAAGCTTATGGCCTCTCTTATC	5' *mnSOD* with *HindIII*
oEL-30	GGGCGGCCGCTTACGCAAAGCGCTT	3' *mnSOD* with *NotI*
oEL-31	GGCATATGCTCCGCGGCCAGACT	5' *pimA* with *NdeI*
oEL-32	TTGGGCCCTCAGTGGTCGTCCTTATCGTTGGA	3' *pimA* with *Bsp120I*

Table S3. *Aspergillus* strains used in this study.

**Strain**	**Genotype**	**Origin/Reference**
A. nidulans		
RJMP 1.59	*pyrG89; pyroA4; veA*$^{+}$	Shaaban et al.[SR1]
rRAW16	*pyrG89; yA2; veA*$^{+}$	Shin et al.[SR2]
TNJ36.1	*pyrG89; pyroA4; pyrG*$^{+}$*; veA*$^{+}$	Kong et al.[SR3]
THS30.3 (control strain)	*pyrG89*; *pyroA*$^{+}$; *veA*$^{+}$; *AfupyrG*$^{+}$	Emri et al.[SR4]
LE1	*pyrG89;ΔaodA::AfupyrG*$^{+}$*; veA*$^{+}$	This study
LE2	*pyrG89;ΔdnmA::AfupyrG*$^{+}$*; veA*$^{+}$	This study
LE3	*pyrG89;ΔmnSOD::AfupyrG*$^{+}$*; veA*$^{+}$	This study
LE4	*pyrG89;ΔpimA::AfupyrG*$^{+}$*; veA*$^{+}$	This study
LE5	*pyroA4; niiA(p)::aodA::pyroA*$^{+}$*; veA*$^{+}$	This study
LE6	*pyroA4; niiA(p)::dnmA::pyroA*$^{+}$*; veA*$^{+}$	This study
LE7	*pyroA4; niiA(p)::mnSOD::pyroA*$^{+}$*; veA*$^{+}$	This study
LE8	*pyroA4; niiA(p)::pimA::pyroA*$^{+}$*; veA*$^{+}$	This study
A. fumigatus		
Af293	Wild type	Brookman & Denning[SR5]

Supplementary References to Table S3

SR1. Shaaban, M. I., Bok, J. W., Lauer, C. & Keller, N. P. Suppressor mutagenesis identifies a velvet complex remediator of *Aspergillus nidulans* secondary metabolism. *Eukaryot Cell* **9,** 1816-1824 (2010).

SR2. Shin, K.S. et al. Differential roles of the ChiB chitinase in autolysis and cell death of *Aspergillus nidulans*. *Eukaryot Cell* **8,** 738-746 (2009).

SR3. Kong, Q. et al Gβ-like CpcB plays a crucial role for growth and development of *Aspergillus nidulans* and *Aspergillus fumigatus*. *PLoS One* 8, e70355 (2013).

SR4. Emri, T. et al. Core oxidative stress response in *Aspergillus nidulans*. *BMC Genomics* **16,** 478 (2015).

SR5. Brookman, J. L. & Denning, D. W. Molecular genetics in *Aspergillus fumigatus*. *Curr Opin Microbiol* **3,** 468-474 (2000).

Table S4 Summary of the observed phenotypes of the *Aspergillus nidulans* mutants tested.

Parameters tested	*Aspergillus nidulans* strains							
	ΔaodA	*aodA*OE	*ΔdnmA*	*dnmA*OE	*ΔmnSOD*	*mnSOD*OE	*ΔpimA*	*pimA*OE
Growth in surface culture	0	0	-	0	0	0	0	0
Oxidative stress tolerance	0	0	0	0	- (to MSB)	0	0	0
Heavy metal tolerance	0	0	0	0	0	0	- (to Cd^{2+})	+ (to Cd^{2+})
Conidiospore viability	-	0	-	0	-	0	-	0
RS production[a] (growing cultures)	+	0	0	-	-	-	+	+
RS production[b] (ageing cultures)	-	0	+	-	0	-	+	+
Stress defense enzyme activity[a]	- (GPx) + (catalase, SOD)	+ (GPx)	+ (GPx, GR, catalase, SOD)	- (GPx) + (catalase)	+ (GR, catalase) - (SOD)	+ (GR) - (catalase)	+ (GPx, GR, catalase)	+ (GR)
Stress defense enzyme activity[b]	- (catalase, SOD)	-	0	-	- (SOD)	- (GPx, catalase, SOD)	+ (catalase)	-
Cell death	0	0	0	0	- (to PAF)	0	0	0
Total respiration rate[a] (growing cultures)	-	0	+	0	0	0	0	-
Total respiration rate[b] (ageing cultures)	0	-	-	0	-	-	-	-
Alternative respiration rate[a] (growing cultures)	-	+	+	0	0	0	+	0

Alternative respiration rate[b] (ageing cultures)	-	+	-	+	-	0	0	0
Mitochondrial morphology	0	0	higher volumetric ratio and number	0	0	0	0	higher volumetric ratio and number
Hyphal diameter	0	-	0	-	0	0	0	-
Sexual development	0	0	0	0	0	0	no ascospores	0
Number of fruiting bodies	+	+	0	0	0	-	0	-
Number of conidiospores	-	0	-	-	-	0	-	-
Autolysis	+ (higher proteinase activity)	0	0	0	0	0	0	0
Sterigmatocystin production	-	-	-	0	+	+	-	+

[a] and [b] - Reactive species (RS) productions[24,27], stress defense enzyme activities[59] and respiration rates[29] measured in growing cultures and in ageing cultures are presented, respectively.
